# Structure-based virtual screening identifies ethyl rosmarinate as a SLC1A6-targeted therapeutic candidate for bladder cancer

**DOI:** 10.3389/fphar.2026.1895264

**Published:** 2026-09-22

**Authors:** Hao Wang, Hongquan Liu, Xin Ma, Wenyu Wang, Jian Ma, Jitao Wu

**Affiliations:** 1 School of Clinical Medicine, Shandong Second Medical University, Weifang, Shandong, China; 2 Department of Urology, The Affiliated Yantai Yuhuangding Hospital of Qingdao University, Yantai, Shandong, China

**Keywords:** bladder cancer, ethyl rosmarinate, SLC1A6, spatial transcriptomics, virtual screening

## Abstract

**Objective:**

SLC1A6 remains poorly characterized in bladder cancer. This study investigated its expression pattern, clinical relevance, cellular localization, functional implications, and potential pharmacological modulation.

**Methods:**

Bulk transcriptomic cohorts, clinical data, single-cell RNA-seq data, and spatial transcriptomic data were analyzed to characterize SLC1A6 in bladder cancer. We evaluated SLC1A6 expression, prognosis, clinicopathological associations, immune microenvironment features, metabolic programs, and predicted drug sensitivity. Single-cell and spatial analyses were used to identify the main cellular source and tissue localization of SLC1A6. Virtual knockout analysis was used to estimate pathway changes after SLC1A6 depletion. SLC1A6 expression was validated using Human Protein Atlas immunohistochemistry images, paired clinical tissue Western blotting, and qPCR/Western blotting in bladder cancer cell lines. Structure-based virtual screening and molecular docking were performed, followed by functional testing of ethyl rosmarinate in T24 and UM-UC-3 bladder cancer cells.

**Results:**

SLC1A6 was upregulated in bladder cancer tissues and cell lines and was associated with poor survival, advanced clinicopathological features, immunosuppressive characteristics, metabolic remodeling, and poorer survival outcomes in immunotherapy-treated cohorts. Single-cell and spatial analyses showed that SLC1A6 was mainly enriched in epithelial tumor cells and epithelial-dominant tumor regions. SLC1A6-high epithelial cells showed enhanced lipid- and glycan-related metabolic activity. Virtual knockout analysis predicted broad pathway alterations after SLC1A6 depletion. Virtual screening prioritized ethyl rosmarinate as a candidate SLC1A6-interacting compound, and CETSA further supported its cellular target engagement with SLC1A6. In T24 cells, ethyl rosmarinate reduced SLC1A6 protein expression and suppressed migration, colony formation, and invasion. Consistent phenotypic effects on migration, clonogenic growth, and invasion were further observed in UM-UC-3 cells.

**Conclusion:**

SLC1A6 is an epithelial-enriched, clinically relevant molecule associated with malignant progression, immune suppression, and metabolic remodeling in bladder cancer. Ethyl rosmarinate may represent a candidate compound for further investigation in SLC1A6-related bladder cancer models.

## Introduction

Bladder cancer is a common urinary system cancer with high recurrence, significant molecular diversity, and minimal long-term survival improvement despite advancements in surgery, chemotherapy, and immunotherapy (Dobruch et al., 2016; Lenis et al., 2020; Kamoun et al., 2020). Recent multi-omics research indicates that metabolic reprogramming is a key feature of urothelial carcinogenesis, significantly contributing to tumor progression, therapy resistance, and immune evasion (Miranda-Gonçalves et al., 2020). Among the metabolic pathways implicated in bladder cancer, amino acid transport and utilization have emerged as critical determinants of tumor cell proliferation, redox homeostasis, and microenvironmental adaptation (Kami Reddy et al., 2024). However, the specific transporters responsible for shaping nutrient availability and metabolic plasticity in bladder cancer remain incompletely understood.

The solute carrier (SLC) superfamily, comprising over 400 membrane transporters, orchestrates transmembrane movement of amino acids, metabolites, ions, and xenobiotics, thereby modulating tumor metabolism and therapeutic responsiveness (Khan et al., 2025). Recent studies have shown that several SLC transporters—including SLC1A5, SLC7A5, and SLC6A14—play indispensable roles in cancer metabolic rewiring and represent emerging therapeutic targets (Cao et al., 2021). The role of SLC1A6, a high-affinity excitatory amino acid transporter, in bladder cancer or any other solid tumor has remained largely unexplored.

Advancements in single-cell RNA sequencing and spatial transcriptomics have highlighted that metabolic gene expression is microenvironment-specific, a detail often missed by bulk sequencing (Wu S. Z. et al., 2021). Spatially resolved profiling enables precise characterization of tumor–stroma interactions and identification of niche-restricted molecular determinants of tumor behavior (Sun et al., 2023). In parallel, reference-based spatial deconvolution algorithms such as RCTD enable the mapping of single-cell reference signatures onto spatial transcriptomic spots, thereby helping to resolve cellular architecture and gene localization in complex tumor tissues (Zaitsev et al., 2022; Kleshchevnikov et al., 2022). Such technologies are particularly valuable in bladder cancer, where tumor cell plasticity and spatial heterogeneity drive distinct therapeutic responses and clinical outcomes.

Given the limited understanding of SLC transporters in bladder cancer and the absence of studies investigating SLC1A6 in oncologic contexts, there is a pressing need to elucidate whether this transporter contributes to tumor biology or clinical progression. In this study, we used differential expression analysis, WGCNA, prognostic modeling, single-cell analysis, and spatial transcriptomics to investigate SLC1A6 in bladder cancer. We focused on its expression pattern, cellular source, spatial localization, clinical relevance, and potential pharmacological modulation. We further validated our findings using Western blotting of paired tumor and normal tissues. Our multi-layered approach uncovers SLC1A6 as a novel tumor-intrinsic molecule potentially involved in bladder cancer progression, suggesting its role as a biomarker or therapeutic target.

## Methods

### Data acquisition and preprocessing

Bladder cancer transcriptome datasets were obtained from public databases. The GSE13507 microarray dataset was obtained from the Gene Expression Omnibus (GEO) on 12 August 2025 (Kim et al., 2010; Lee et al., 2010). The raw CEL files underwent normalization using the affy and limma R packages, including background correction, log2 transformation, and quantile normalization (Ritchie et al., 2015). TCGA-BLCA RNA-seq profiles and matched clinical annotations were retrieved through the UCSC Xena platform (Xu et al., 2024). Genes with counts per million below 1 in more than 50% of samples were excluded before analysis. Unless otherwise noted, analyses were performed using R version 4.3.2.

### Differential expression analysis

Differentially expressed genes (DEGs) between tumor and normal bladder tissues were identified independently in the two cohorts. For microarray-based datasets, gene-level differences were estimated with the limma empirical Bayes framework. For RNA-seq data, differential expression was assessed using the DESeq2 package (Love et al., 2014). Genes were defined as DEGs when meeting both criteria: |log_2_ fold change| ≥ 1, false discovery rate (FDR) < 0.05.

### Gene co-expression network construction and module–trait analysis

The WGCNA package was utilized to construct a co-expression network for identifying gene modules linked to bladder cancer status (Langfelder and Horvath, 2008). Only genes ranked within the highest quartile of expression variance were included in the co-expression network. Samples underwent hierarchical clustering to eliminate potential outliers, followed by selecting β based on the scale-free topology fit criterion, aiming for an *R* ^2^ exceeding 0.8. The adjacency relationships were transformed into topological-overlap measures, and genes were then clustered according to their network similarity. Modules were initially defined using dynamic tree cutting with a minimum of 100 genes, and those with highly similar eigengene profiles were merged at a cut height of 0.25. Module eigengenes were then correlated with tumor or normal status by Pearson correlation analysis, and modules showing significant phenotype associations after correction for multiple testing were retained. To prioritize robust bladder cancer-related candidates, we integrated three evidence layers, including DEGs from GSE13507, DEGs from TCGA-BLCA, and genes from tumor-associated WGCNA modules. The overlapping genes were identified using VennDiagram and dplyr in R, resulting in a candidate gene set corroborated by both differential-expression analysis and co-expression network evidence (Chen and Boutros, 2011).

### Protein–protein interaction network analysis

Protein-level interactions among the overlapping candidate genes were investigated using STRING (Szklarczyk et al., 2023). The analysis was restricted to *Homo sapiens*, and interactions with a minimum confidence score of 0.40 were retained. The network was refined by removing disconnected nodes and was visualized to provide an overview of the interaction landscape among the overlapping candidate genes. SLC1A6 was selected for focused downstream analysis based on combined evidence from tumor-associated expression patterns, biological interpretability as a solute carrier/glutamate transporter, feasibility for downstream validation and virtual screening, and limited prior characterization in bladder cancer.

### Functional enrichment

The overlapping candidate genes obtained from the integrative screening were subjected to KEGG pathway enrichment analysis using clusterProfil (Yu et al., 2012; Kanehisa and Goto, 2000; Kanehisa et al., 2023). Gene symbols were annotated with org.Hs.eg.db, and pathway overrepresentation was evaluated using a hypergeometric testing framewor (Wang et al., 2022). Pathways with adjusted p values below 0.05 were considered statistically enriched. Enrichment results, including gene ratio, rich factor, gene count, and adjusted p value, were displayed as bar plots and bubble plots using clusterProfiler, ggplot2, and enrichplot (Wu T. et al., 2021). To further summarize pathway classification, KEGG terms were visualized with a circlize-based circular plot, which integrated functional category, gene number, enrichment statistics, and pathway classification information into a multi-layer layout.

### Expression pattern analysis

Normalized TCGA-BLCA RNA-seq values were extracted and converted into Z-scores for heatmap visualization to compare the expression profiles of overlapping candidate genes. Heatmaps were generated with pheatmap, and hierarchical clustering was performed using Euclidean distance with complete-linkage aggregation (Zhang X. et al., 2023). Pathological T, N, and M classifications, overall pathological stage, and smoking status were added as annotation tracks to relate gene-expression patterns to clinical phenotypes. The same candidate gene set was then evaluated in the GSE13507 cohort after platform-specific normalization. For cross-platform comparability, the GSE13507 expression matrix was also Z-score transformed and visualized using the same clustering strategy.

### Survival analysis based on SLC1A6 expression

The prognostic significance of SLC1A6 was assessed in the TCGA-BLCA cohort using endpoints such as overall survival, progression-free interval, and disease-specific survival. Clinical survival annotations and normalized RNA-seq expression profiles were obtained from UCSC Xena. Patients were categorized into SLC1A6-high and SLC1A6-low groups based on the median SLC1A6 expression level. Kaplan–Meier curves were generated using the survival package, and group differences were evaluated with the log-rank test (Lee et al., 2020). Cox proportional hazards regression was used to estimate hazard ratios, and survival curves were visualized with survminer.

### Differential expression analysis in TCGA and GSE13507

SLC1A6 expression was compared between bladder tumor and normal tissues in TCGA-BLCA using normalized log2-transformed TPM values. Group differences were displayed with ggplot2-based boxplots and tested using the Wilcoxon rank-sum test. The expression pattern of SLC1A6 was further validated in GSE13507, in which the processed microarray matrix was analyzed according to the normalization procedure described above. Tumor–normal comparisons in GSE13507 were visualized and statistically tested using the same strategy to maintain consistency between datasets.

### Pan-cancer expression analysis in TCGA and TCGA + GTEx

The expression distribution of SLC1A6 across various cancer types was analyzed using RNA-seq data from the TCGA Pan-Cancer Atlas, obtained via UCSC Xena. For each available tumor type, tumor samples and corresponding matched or unmatched normal tissues were extracted when available. Violin plots generated with ggplot2 illustrated SLC1A6 expression distributions, and the Wilcoxon rank-sum test evaluated tumor-normal differences. To increase the number of normal tissue references, TCGA tumor samples were compared with GTEx normal tissues using the uniformly processed TCGA–GTEx RNA-seq dataset available through the UCSC Xena Toil Recompute Compendium. Rather than independently combining expression matrices processed by different pipelines, we used the Toil-recomputed data in which TCGA and GTEx RNA-seq samples had been reprocessed using a common computational workflow for read alignment and gene-expression quantification, thereby reducing computational batch effects arising from differences in the original processing pipelines. Gene-expression values from the harmonized matrix were used on the same expression scale for tumor–normal comparisons, and no additional batch-correction algorithm was applied after retrieval. Tumor and corresponding normal tissue groups were then compared using the Wilcoxon rank-sum test (GTEx Consortium, 2013).

### Pan-cancer prognostic analysis of SLC1A6

Pan-cancer survival analysis was performed using harmonized TCGA clinical and RNA-seq datasets obtained from UCSC Xena. Four survival outcomes were analyzed, including overall survival, progression-free interval, disease-free interval, and disease-specific survival. Within each cancer type, patients were classified into high- and low-SLC1A6 groups using the median SLC1A6 expression value as the cutoff. Univariate Cox proportional hazards models were fitted with the survival package to calculate hazard ratios, 95% confidence intervals, and p values. Forest plots generated with forestplot and ggplot2 were used to summarize the prognostic effect of SLC1A6 across tumor types and survival endpoints. Cancer types without sufficient clinical follow-up information were excluded from the corresponding analysis.

### Drug sensitivity correlation analysis

Drug-sensitivity profiles for bladder cancer cell lines were retrieved from GDSC and CTRP through the oncoPredict and pRRophetic pipelines (Yang et al., 2013; Zhang H. et al., 2023; Wang et al., 2023a; Maeser et al., 2021). Using TCGA-BLCA expression profiles as input, predicted IC50 values were inferred by ridge regression-based models. Log2-transformed TPM values of SLC1A6 from TCGA-BLCA were analyzed using Pearson correlation to assess the relationship between SLC1A6 expression and the estimated IC50 values of anticancer agents. The stats package was used to calculate correlation coefficients and p-values, while ggplot2 generated regression plots with 95% confidence intervals. Only compounds with complete predicted IC50 values across samples were retained for final analysis.

### Genomic alteration and expression association analysis

TCGA pan-cancer CNV and SNV profiles were obtained from UCSC Xena for evaluating the relationship between genomic alterations and SLC1A6 expression (Dong et al., 2020). For CNV analysis, GISTIC2.0 thresholded scores were grouped into copy-number loss, neutral status, and copy-number gain according to values below, equal to, or above zero. For SNV analysis, MAF files were summarized at the gene level, and samples carrying at least one non-synonymous SLC1A6 mutation were classified as Mut, whereas the remaining samples were classified as WT. Matched normalized RNA-seq expression values were then extracted for the same samples. The Kruskal–Wallis test with Dunn’s *post hoc* correction was used to evaluate SLC1A6 expression differences among CNV groups, and the Wilcoxon rank-sum test was employed for comparisons between Mut and WT groups. Violin plots generated with ggplot2 were used to show expression distributions across cancer types.

### Clinicopathologic grade and metastatic status analysis

Clinicopathologic annotations, including histologic grade and AJCC M stage, were collected from TCGA clinical records (Lechner et al., 2022). Tumor grade was standardized into four categories, G1 to G4, according to the original pathological information, and cases with unavailable or unclear grade annotations were removed. Distant metastatic status was defined as M0 for patients without distant metastasis and M1 for those with documented distant metastasis. In cancer types with sufficient sample numbers, SLC1A6 expression was compared across grade categories using the Kruskal–Wallis test and between M0 and M1 groups using the Wilcoxon rank-sum test. The corresponding expression distributions were displayed using violin plots.

### Microsatellite instability, tumor mutation burden, and stemness index correlation analysis

Pan-cancer MSI, TMB, and stemness score matrices were retrieved from published computational resources integrated in UCSC Xena (Wang et al., 2023b; Yamamoto et al., 2024). Stemness-related indices included DNA methylation-based stemness scores and RNA expression-based stemness scores. SLC1A6 expression values were matched with these indicators according to sample identifiers within each cancer type. Pearson correlation analysis was then performed to quantify the association between SLC1A6 expression and each genomic instability or stemness-related metric. Correlation coefficients and two-sided p values were calculated in R, and the results were summarized as bar plots to show both the direction and magnitude of the associations.

### Immune checkpoint–related gene correlation analysis

A literature-derived immune regulatory gene set was assembled, covering stimulatory checkpoints, inhibitory checkpoints, chemokines, chemokine receptors, and MHC-related genes (Kalbasi and Ribas, 2020). Normalized TCGA expression matrices were used to calculate pairwise Pearson correlations between SLC1A6 and each immune-related gene across cancer types. The resulting correlation matrix was visualized using pheatmap, with triangular color annotations indicating positive or negative correlation patterns.

### Diagnostic performance evaluation using ROC analysis

ROC analysis assessed the diagnostic significance of SLC1A6 in cancer types with accessible tumor and normal samples. The analysis was performed with the pROC package (Robin et al., 2011). AUC values with 95% confidence intervals were computed to assess SLC1A6’s efficacy in differentiating tumor tissues from normal controls. ROC curves were drawn using ggplot2, and representative cancer types with clear diagnostic performance were selected for visualization.

### Immune cell infiltration estimation using xCell

Immune and stromal infiltration patterns were estimated using xCell, which infers the enrichment of 64 immune and stromal cell signatures from bulk transcriptomic data (Angel et al., 2025). Normalized log2 TPM expression profiles from TCGA pan-cancer cohorts were used as the input matrix. Pearson correlation analysis was conducted to assess the relationship between SLC1A6 expression and xCell-derived cell-type scores across various cancer types. Triangular heatmaps, created using pheatmap and ggplot2, were used to present correlation coefficients and p-values.

### Association with immunophenoscore

Immunophenoscore data for TCGA cancer types were obtained from The Cancer Immunome Atlas (Charoentong et al., 2017). IPS components, including effector cells, suppressor cells, antigen-presenting machinery, and checkpoint or immune-modulator scores, were merged with SLC1A6 expression profiles according to sample identifiers. Pearson correlation analysis was performed for each IPS component within each cancer type, and the resulting associations were visualized using triangular correlation heatmaps.

### Immune population abundance estimation using MCPcounter

MCPcounter was employed to estimate the abundance of key immune and stromal populations, such as T cells, myeloid cells, and fibroblasts (Zhu et al., 2024). MCPcounter scores were matched with SLC1A6 expression levels across TCGA cancer types. Pearson correlation analysis was then used to examine associations between SLC1A6 and each MCPcounter-derived population. Triangular heatmaps were generated to summarize correlation patterns across cancer types.

### Correlation between SLC1A6 expression and clinicopathologic features in bladder cancer

For bladder cancer-specific clinical analysis, TCGA-BLCA clinical annotations and RNA-seq expression profiles were retrieved from UCSC Xena. Clinical variables included pathological T, N, and M categories, AJCC overall stage, primary therapy outcome, radiotherapy status, age at diagnosis, and molecular subtype classification. SLC1A6 expression values were extracted from the harmonized RNA-seq matrix and compared across categorical clinicopathologic groups. Wilcoxon rank-sum tests were used for two-group comparisons, whereas Kruskal–Wallis tests followed by Dunn’s *post hoc* correction were applied for variables with three or more groups. Violin plots were used to display expression distributions, with mean and standard error values shown for each group. To further evaluate the relationship between SLC1A6 expression and muscle-invasive status, an additional stratified analysis was performed in the TCGA-BLCA cohort. Because the available non-muscle-invasive cases in TCGA-BLCA were represented by pathologic T1 tumors, T1 tumors were categorized as the available NMIBC subgroup, whereas T2–T4 tumors were categorized as MIBC. SLC1A6 expression between the two groups was compared using the Wilcoxon rank-sum test.

### Cox regression analysis in bladder cancer

Clinical variables and survival information for TCGA-BLCA were downloaded from UCSC Xena. Univariate Cox proportional hazards regression was performed to evaluate the association of SLC1A6 expression and clinicopathological variables with overall survival. Hazard ratios and 95% confidence intervals were calculated. Because SLC1A6 expression was not significantly associated with overall survival in the univariate Cox model, it was not further interpreted as an independent prognostic factor (Latour et al., 2024).

### Single-cell RNA-seq analysis

Single-cell RNA-seq datasets of urothelial carcinoma, including GSE130001, GSE222315, and GSE135337, were obtained from GEO. Raw expression matrices were processed using Seurat v4.3. Low-quality cells were excluded according to gene counts, UMI counts, and mitochondrial transcript proportions. After quality control, the three datasets were normalized and integrated to construct a unified bladder cancer single-cell atlas. Dimensionality reduction was performed by PCA, followed by graph-based clustering and t-SNE or UMAP visualization. Major cell lineages were annotated according to canonical marker genes, including epithelial cells, fibroblasts, endothelial cells, myofibroblasts, macrophages, T cells, B cells, and plasma cells. SLC1A6 expression was then examined across clusters and annotated cell types using feature plots and cell-type-level summaries (Wang et al., 2020; Mangiola et al., 2021; Colwill and Gräslund, 2011).

To further evaluate SLC1A6-associated metabolic programs, single-cell pathway activity scores were calculated for selected metabolic gene sets and compared among SLC1A6-defined epithelial subgroups. In addition, scTenifoldKnk-based virtual knockout analysis was used to predict transcriptional and pathway-level alterations after simulated SLC1A6 depletion, and the affected genes and pathways were summarized for downstream interpretation.

### Immunohistochemistry validation

SLC1A6 protein expression in bladder tissues was evaluated using immunohistochemistry images from the Human Protein Atlas (Gouin et al., 2021). Representative images of normal bladder urothelium and urothelial carcinoma were examined. Staining intensity, subcellular distribution, and tumor–normal differences were interpreted according to HPA annotation criteria.

### Subcellular localization analysis

Subcellular localization information for SLC1A6 was obtained from the HPA Cell Atlas module. Two representative human cell lines, A-431 and HAP1, were selected based on the availability of high-resolution confocal microscopy images. Nuclear staining was visualized with DAPI, while SLC1A6 protein localization was assessed using the corresponding antibody signal. Protein distribution patterns, including membrane, cytoplasmic, and vesicular localization, were interpreted according to HPA annotation standards.

### Spatial transcriptomics analysis

Spatial transcriptomic analysis was performed using the 10× Genomics Visium bladder cancer dataset GSE246011. RCTD was used as the spatial deconvolution algorithm throughout this study (Abdel-Hafiz et al., 2023; Wolf et al., 2018). One representative tumor section was selected for downstream analysis. Raw sequencing outputs were processed with the 10× Genomics Space Ranger pipeline to generate spot-level count matrices and matched histological images. The filtered matrix was imported into Seurat for quality control, normalization, and visualization. Spots with low gene detection, low UMI counts, excessive mitochondrial transcript proportions, or other outlier characteristics were excluded, and the remaining spots were normalized and log-transformed (César-Razquin et al., 2015). For spatial deconvolution, we used the integrated single-cell reference constructed from three bladder cancer scRNA-seq datasets. The three datasets were integrated after standard quality control and Harmony-based batch correction. Integration quality was assessed by the degree of overlap among datasets after correction, while cell-type identities were assigned according to canonical lineage markers and confirmed by marker-expression dot plots. This annotated atlas was then used as the reference for RCTD. The resulting integrated scRNA-seq atlas included major cell populations such as epithelial cells, fibroblasts, endothelial cells, macrophages, T cells, B cells, plasma cells, and other stromal/immune subsets. The annotated integrated single-cell atlas was used as the reference input for RCTD, and the Visium spot-level count matrix was used as the spatial query. RCTD was performed to estimate the cellular composition of each spatial spot and to assign dominant cell types across the tissue section. The inferred spatial distribution of major cell populations was then visualized to characterize the microenvironmental architecture. In addition, the spatial expression of SLC1A6 was mapped onto the tissue section using normalized Visium matrices to determine its localization relative to epithelial and non-epithelial niches.

### Structure-based virtual screening and molecular docking targeting SLC1A6

To identify candidate compounds with potential interaction capacity toward SLC1A6, a structure-guided virtual screening workflow was performed using Schrödinger Suite 2023-1. A publicly available human SLC1A6 protein structure/model was used as the receptor for docking analysis. The receptor structure was prepared using the Protein Preparation workflow. Hydrogen atoms were added, bond orders and protonation states were assigned, and restrained energy minimization was performed. The receptor was prepared under physiological pH conditions, with the preprocessing pH set to 7.4.

Potential ligand-accessible pockets were predicted using SiteMap with default parameters. Pocket filtering was performed according to pocket size, accessibility, and physicochemical properties. The minimum pocket volume threshold was set to 100 Å^3^, and the probe radius was set to 1.0 Å. The selected ligand-accessible pocket was used to generate the Glide docking grid. The docking grid was centered at x = 3.625, y = 8.392, and z = −0.647. The outer grid box size was set to 30 × 30 × 30 Å^3^, and the inner ligand box size was set to 10 × 10 × 10 Å^3^. The L6000 Natural Product Library for HTS, containing 4,566 natural compounds, was used as the screening library. Ligands were prepared using the LigPrep module to generate reasonable ionization states, stereoisomers, tautomers, and low-energy conformations, and structurally unsuitable conformations were removed. After ligand preparation, 14,194 ligand structures were generated. QikProp was then used to evaluate ADME/T-related properties, drug-likeness, and chemical-space properties, and 5,682 ligand structures were retained for docking. A hierarchical Glide docking workflow was performed, including high-throughput virtual screening (HTVS), standard precision (SP), and extra precision (XP) docking. In the HTVS stage, the top 15% of candidates ranked by docking score were retained, yielding 852 structures. In the SP stage, the top 20% of candidates were retained, yielding 170 structures. XP docking was then performed for the remaining top-ranked candidates, and 132 top-ranked candidates were retained for subsequent prioritization. Compounds were ranked primarily according to docking score, with more negative scores indicating stronger predicted binding affinity. To further prioritize candidate compounds, the top-ranked docking candidates were subjected to Prime MM/GBSA binding free-energy estimation. MM/GBSA calculations were performed using the VSGB2.1 implicit solvent model and the S-OPLS force field in Schrödinger, with dielectric and other calculation parameters set according to the default Prime MM/GBSA workflow. Candidate compounds were finally prioritized based on an integrated assessment of docking score, MM/GBSA ΔGbind, predicted binding pose, and key residue interactions.

### Clinical tissue collection and protein validation

Bladder tumor tissues and matched adjacent non-tumor tissues were collected from nine patients who provided informed consent prior to surgery. The Institutional Review Board of The Affiliated Yantai Yuhuangding Hospital of Qingdao University approved the use of these clinical specimens under clinical trial number K2026-033, with expedited approval on 19 January 2026. Patient enrollment was conducted from 1 December 2025 to 19 January 2026. Proteins were extracted from tissue using RIPA lysis buffer supplemented with a protease inhibitor cocktail. Equivalent protein amounts were separated using SDS-PAGE and then transferred to PVDF membranes. Membranes were blocked with 5% skimmed milk and incubated overnight at 4 °C with an anti-SLC1A6 primary antibody, followed by exposure to an HRP-conjugated secondary antibody. β-Actin served as the reference protein for loading normalization. Chemiluminescent signals were captured, and the corresponding band densities were measured with ImageJ. The relative expression of SLC1A6, normalized to β-Actin, was plotted using GraphPad Prism.

### Cell lines and expression validation

SLC1A6 expression was validated at the cellular level using one non-malignant urothelial cell line and four bladder cancer cell lines: SV, EJ, 253J, T24, and UM-UC-3. Cells were cultured in a medium with 10% fetal bovine serum and 1% penicillin-streptomycin, incubated at 37 °C with 5% CO_2_ humidity. For qPCR analysis, cellular RNA was isolated with TRIzol and converted into cDNA using a commercial reverse-transcription kit. Real-time quantitative PCR was performed using SYBR Green chemistry. The SLC1A6 primer sequences were: forward, 5′-AAC​ACA​AGG​GGC​AGA​GTC​CTC​TCA​G-3′; reverse, 5′-CCA​GCA​ATC​AGG​GAA​CAG​GAT​GC-3′. GAPDH was used as the housekeeping reference gene. Relative SLC1A6 transcript levels were calculated using the 2^−ΔΔCt method, with SV cells used as the calibrator. Three independent experiments were performed for each cell line. Primer amplification efficiency was validated using five-point serially diluted cDNA standard curves, and primers with amplification efficiencies between 90% and 110% and *R* ^2^ values greater than 0.99 were considered acceptable. Proteins from the specified cell lines were lysed in RIPA buffer with protease inhibitors for Western blotting. Protein lysates underwent SDS-PAGE electrophoresis followed by transfer to PVDF membranes. After blocking with milk, membranes were incubated overnight at 4 °C with the SLC1A6 primary antibody, followed by an HRP-conjugated secondary antibody. Chemiluminescence was used to detect immunoreactive bands, with β-Actin serving as the normalization control.

### ER treatment in T24 cells

Ethyl rosmarinate (ER; used here only as the abbreviation for ethyl rosmarinate), the highest-ranked compound from structure-guided screening and docking analysis, was selected for functional testing in T24 cells. ER stock solution was prepared and serially diluted to the required working concentrations before cell exposure. Based on the 24-, 48-, and 72-h viability curves in both T24 bladder cancer cells and SV-HUC-1 normal urothelial cells, 5 μM ER was selected as the working concentration for subsequent phenotypic assays. This concentration was below the calculated IC50 values in both cell lines at all tested time points and produced a measurable inhibitory effect in T24 cells while avoiding excessive cytotoxicity in normal urothelial cells. Vehicle-treated cells processed in parallel served as the control group. To further evaluate whether the phenotypic effects of ER were reproducible in an additional bladder cancer cell line, UM-UC-3 cells were included for independent functional validation. A preliminary dose-response viability assay was performed using ER concentrations of 0, 0.625, 1.25, 2.5, 5, 10, 20, 40, and 80 μM. Based on the limited reduction in cell viability observed at 5 μM, this concentration was used for subsequent UM-UC-3 functional assays to minimize confounding by overt cytotoxicity. Vehicle-treated UM-UC-3 cells were processed in parallel as controls.

### Cell viability assay

Cell viability after ER exposure was measured using a CCK-8 assay. T24 bladder cancer cells and SV-HUC-1 normal urothelial cells were seeded in 96-well plates and incubated overnight for attachment. Cells were then treated with ER at 0, 0.625, 1.25, 2.5, 5, 10, 20, 40, or 80 μM for 24, 48, or 72 h. At each indicated time point, CCK-8 reagent was added according to the manufacturer’s instructions, and absorbance was measured using a microplate reader. Cell viability was normalized to the untreated control group at the same time point. Dose-response curves were fitted using nonlinear regression with non-zero concentrations, and IC50 values were calculated to evaluate the time-dependent inhibitory effect of ER. The selectivity index was calculated as IC50(SV-HUC-1 cells)/IC50(T24 cells). In addition, UM-UC-3 cells were exposed to ER at 0, 0.625, 1.25, 2.5, 5, 10, 20, 40, or 80 μM for 24 h to evaluate cell viability and support selection of the working concentration for the second-cell-line validation experiments.

### Western blot analysis after ER treatment

To determine whether ER altered SLC1A6 protein abundance, T24 cells were exposed to 5 μM ER or vehicle. Following treatment, total cellular protein was isolated using protease inhibitor-containing RIPA buffer. Equal amounts of protein were separated by SDS-PAGE and transferred onto PVDF membranes. Blocked membranes were incubated with antibodies against SLC1A6 and β-Actin, followed by incubation with an HRP-conjugated secondary antibody. Chemiluminescent bands were detected, and SLC1A6 protein levels were quantified by densitometry after β-Actin normalization.

### Wound healing assay

The effect of ER on bladder cancer cell migration was assessed using a scratch-wound assay in T24 and UM-UC-3 cells. T24 cells were plated and cultured until a confluent monolayer was formed. A linear wound was generated using a sterile pipette tip, followed by gentle PBS washing to remove detached cells. Cells were then cultured in medium containing 5 μM ER or vehicle control. Microscopic images of the wound area were taken at 0 and 24 h. The wound healing rate was calculated by comparing the wound width or wound area at the indicated time points. For the UM-UC-3 validation experiment, cells were treated with 5 μM ER or vehicle and imaged at 0 and 24 h using the same analytical procedure. The 24-h time point was selected as the predefined endpoint to capture measurable short-term wound closure while avoiding excessive closure at later time points and limiting the increasing contribution of prolonged cell proliferation to the migration readout.

### Colony formation assay

Colony formation assays were performed in T24 and UM-UC-3 cells to assess clonogenic growth following ER treatment. T24 cells were seeded into culture plates at low density and treated with 5 μM ER or vehicle control. Cells were cultured until colonies became visible. Colonies were fixed and stained using crystal violet. Colony images were captured to quantify colony numbers, assessing ER’s impact on the clonogenic growth of bladder cancer cells.

### Transwell invasion assay

The invasive potential of T24 and UM-UC-3 cells was evaluated using Transwell invasion assays. T24 cells were seeded into the upper chamber of Transwell inserts after treatment with either 5 μM ER or a vehicle control. The lower chamber contained a medium with chemoattractant. Post-incubation, cells on the membrane’s upper surface were removed, while those that penetrated the membrane were fixed and stained. Representative images were obtained under a microscope, and invaded cells were counted in multiple randomly selected fields. For UM-UC-3 cells, invaded cells were quantified using the same biological-replicate and image-averaging strategy described above.

### Statistical analysis

Unless otherwise stated, *in vitro* experiments were performed using three independent biological replicates. Independent biological replicates were defined as experiments performed with separately cultured cells on different experimental days or from independent cell passages. Technical wells, repeated image measurements, or microscopic fields obtained within the same experiment were averaged to generate one value for each biological replicate and were not treated as independent biological replicates. Quantitative data are presented as mean ± standard deviation, with individual data points shown where applicable. Student’s t-test was used for two-group comparisons, while one-way ANOVA followed by an appropriate *post hoc* test was applied for multiple-group comparisons. Statistical significance was defined as P < 0.05.

### Cellular thermal shift assay

Cellular thermal shift assay (CETSA) was performed to evaluate the cellular target engagement between ethyl rosmarinate and SLC1A6. T24 cells were treated with 5 μM ethyl rosmarinate or DMSO control, harvested, and divided into aliquots. The samples were heated at the indicated temperature gradient, followed by cooling and protein extraction. Soluble protein fractions were collected after centrifugation and analyzed by Western blotting using an anti-SLC1A6 antibody. The relative SLC1A6 band intensity at each temperature was normalized and compared between the ethyl rosmarinate-treated and DMSO-treated groups.

## Results

### Differential expression analysis in two independent cohorts

Table 1 presents the datasets utilized in this study. Differential expression analyses of the GSE13507 and TCGA-BLCA datasets revealed clear transcriptional differences between tumor and normal bladder tissues. In GSE13507, a distinct set of significantly upregulated and downregulated genes was observed (Figure 1A), while the TCGA-BLCA cohort displayed a comparable differential expression profile with notable divergence in gene distribution (Figure 1B). These findings indicate consistent transcriptomic alterations across microarray and RNA-seq platforms and provide a robust basis for downstream integrative analyses.

**TABLE 1:** Summary of cohorts included in this study.

Cohort	Sample size	Description
GSE13507	256	Bulk transcriptomic cohort
GSE154261	99	Bulk RNA-seq cohort of NMIBC
GSE19423	48	BCG-treated bladder cancer cohort
GSE37815	24	Bladder cancer expression cohort
GSE39281	93	Metastatic bladder cancer cohort
GSE48075	142	Molecular subtype cohort
GSE48276	128	FFPE bladder tumor cohort
GSE52219	23	Neoadjuvant chemotherapy cohort
GSE69795	61	Chemotherapy-related urothelial cohort
GSE70691	49	MVAC-treated bladder cancer cohort
IMvigor210	248	Immunotherapy cohort
TCGA-BLCA	412	TCGA bladder cancer cohort
GSE246011	2	Spatial transcriptomics cohort
GSE130001	2	Single-cell RNA-seq cohort
GSE222315	13	Single-cell RNA-seq cohort
GSE135337	8	Single-cell RNA-seq cohort
Lauss cohort 2017 (CAR-T/ACT)	27	Exploratory immunotherapy cohort

**FIGURE 1:**
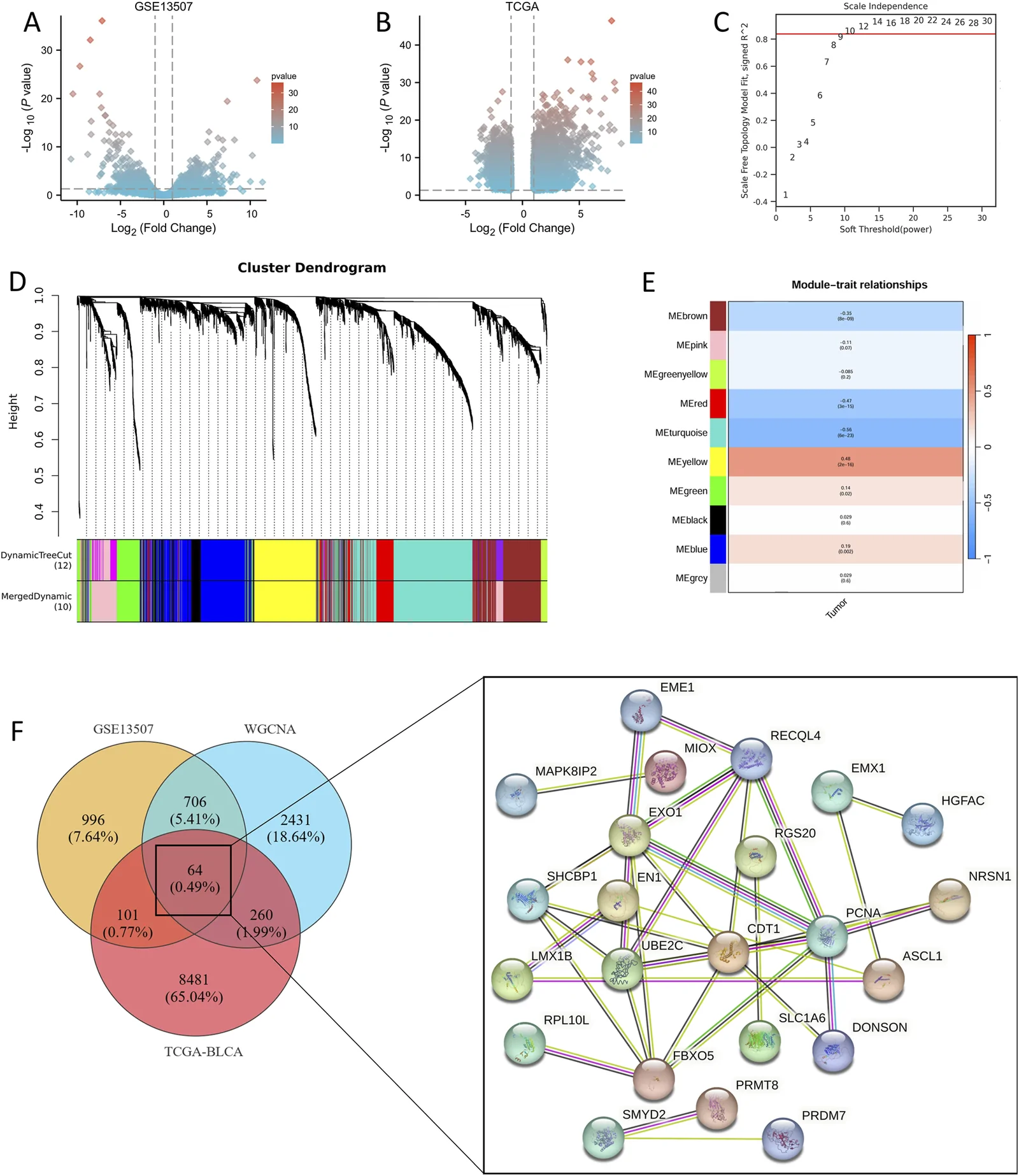
Identification of robust bladder cancer–related genes through differential expression analysis, WGCNA, and PPI network construction. **(A, B)** Volcano plots showing the distribution of differentially expressed genes (DEGs) between tumor and normal bladder tissues in the GSE13507 and TCGA-BLCA cohorts. Significantly upregulated and downregulated genes are highlighted. **(C)** Determination of the optimal soft-thresholding power (β) for WGCNA based on the scale-free topology criterion. **(D)** Hierarchical clustering dendrogram of variance-filtered genes with corresponding dynamic tree cut and merged modules. **(E)** Module–trait correlation heatmap demonstrating the relationship between module eigengenes and tumor status. **(F)** Intersection of DEGs from GSE13507, DEGs from TCGA-BLCA, and tumor-associated WGCNA modules, alongside the STRING-derived protein–protein interaction network of overlapping genes, alongside the STRING-derived protein–protein interaction network of overlapping genes.

### Construction of co-expression modules and identification of tumor-associated modules

A weighted co-expression network was developed to further analyze transcriptomic organization. The soft-thresholding power required for approximation of scale-free topology was determined from the β–scale-free curve (Figure 1C). Hierarchical clustering identified multiple modules of co-expressed genes (Figure 1D), each representing distinct transcriptional programs. Correlation analysis between module eigengenes and clinical traits demonstrated that several modules (yellow, blue, green) were strongly associated with tumor status (Figure 1E). These tumor-related modules were selected for subsequent gene intersection and biological prioritization.

### Integration of multi-cohort evidence and identification of key intersecting genes

To obtain high-confidence candidate genes implicated in bladder cancer, we intersected DEGs from GSE13507, DEGs from TCGA-BLCA, and genes from tumor-associated WGCNA modules. This integrative strategy yielded a shared set of overlapping genes supported by independent datasets and co-expression structures (Figure 1F, left). These intersecting genes likely represent core transcriptional drivers of bladder cancer biology.

### Protein–protein interaction network identifies key hub genes

The intersecting gene set was further analyzed using the STRING database to construct a protein–protein interaction network. The resulting PPI structure displayed strong interconnectivity among candidate genes and highlighted several nodes with high interaction degrees (Figure 1F, right). These hub genes may serve as central regulators or potential biomarkers in bladder cancer progression.

### Functional enrichment

KEGG enrichment analysis of the intersecting gene set revealed significant enrichment in multiple biological pathways, with a marked concentration in DNA repair–related processes and metabolic regulation. Among the top-ranked pathways, mismatch repair exhibited the highest gene contribution, followed by lysine degradation, inositol phosphate metabolism, and several cancer-related pathways including gastric cancer, breast cancer, and antifolate resistance (Figure 2A). Additional pathways involving cellular signaling—such as the MAPK signaling pathway, calcium signaling pathway, and Hippo signaling pathway—were also represented within the enriched set. These findings were corroborated by the bubble-plot visualization, which highlighted pathways with both high rich factors and strong statistical significance (Figure 2D). A circos-based classification further demonstrated that enriched pathways were broadly distributed across KEGG categories, primarily spanning genetic information processing, metabolism, and human disease modules, with distinct clusters reflecting the biological diversity of the associated genes (Figure 2C). It should be noted that this enrichment analysis was performed on the intersecting candidate gene set obtained from differential expression and WGCNA screening, rather than on SLC1A6 alone. These enriched pathways collectively reflect the diverse biological programs represented by the intersecting candidate gene set. Mismatch repair may reflect alterations in DNA repair and genomic maintenance, whereas lysine degradation may indicate changes in amino acid metabolism and related epigenetic regulation. These findings were considered collectively and were not interpreted as direct evidence that SLC1A6 itself regulates any single enriched pathway.

**FIGURE 2:**
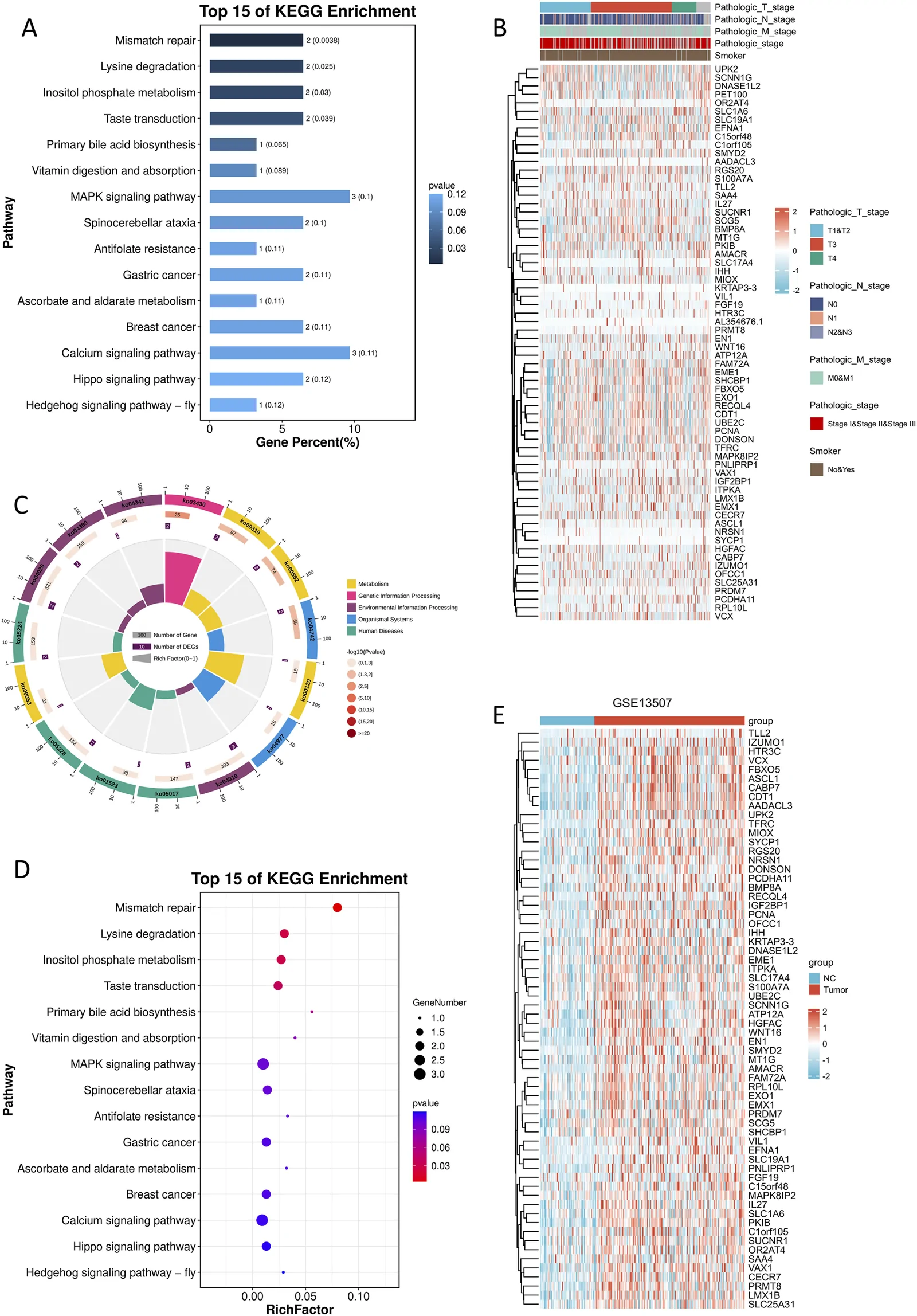
Functional enrichment and expression profiling of intersecting candidate genes. **(A)** Bar plot of the top 15 KEGG enrichment pathways, showing gene ratios and adjusted p values. **(B)** Heatmap of the intersecting gene expression profiles in the TCGA-BLCA cohort, with clinical annotations for pathological T stage, N stage, M stage, composite pathological stage, and smoking status. **(C)** Circos plot summarizing KEGG pathway classifications, gene numbers, and enrichment metrics grouped by major functional categories. **(D)** Bubble plot of the top 15 KEGG pathways, displaying rich factors, gene numbers, and significance levels. **(E)** Heatmap of gene expression validation in the GSE13507 dataset comparing tumor and normal tissues.

### Cross-cohort expression profiling identifies consistent tumor-associated expression patterns

Expression profiling of the intersecting genes in the TCGA-BLCA cohort revealed clear differences between tumor and normal samples, with many genes displaying coherent clustering patterns aligned with biological or clinical phenotypes (Figure 2B). Distinct blocks of gene expression were associated with pathological T stage, N stage, M stage, composite pathological stage, and smoking status, indicating that several of these genes may reflect key clinical characteristics or tumor progression features. To validate these observations, the same gene set was assessed in the GSE13507 cohort. Consistent with TCGA findings, the expression landscape exhibited highly similar tumor-associated patterns, with a reproducible separation of tumor and normal samples (Figure 2E). Several genes demonstrated stable upregulation or downregulation across both datasets, confirming the robustness of transcriptional signatures derived from the intersecting candidate genes.

### SLC1A6 was significantly upregulated in bladder cancer and validated at both tissue and cellular levels

We first assessed the expression pattern of SLC1A6 in public bladder cancer cohorts. In both the GSE13507 and TCGA datasets, SLC1A6 expression was significantly higher in tumor tissues than in normal bladder tissues (Figures 3D,E). To further validate this finding at the protein level, immunohistochemical staining from the HPA database revealed stronger SLC1A6 staining in urothelial carcinoma tissues compared to normal bladder tissues (Figure 3G). Consistently, Western blot analysis of paired clinical samples confirmed elevated SLC1A6 protein expression in tumor tissues, which was further supported by quantitative densitometric analysis (Figures 3I–K). In addition, qPCR analysis showed that SLC1A6 mRNA expression was increased in bladder cancer cell lines compared with normal urothelial cells, with the most prominent expression observed in T24 cells (Figure 3F). Western blotting further confirmed the differential protein expression pattern of SLC1A6 across bladder cancer cell lines (Figure 3H). Together, these results demonstrated that SLC1A6 is aberrantly overexpressed in bladder cancer at both the transcriptomic and protein levels.

**FIGURE 3:**
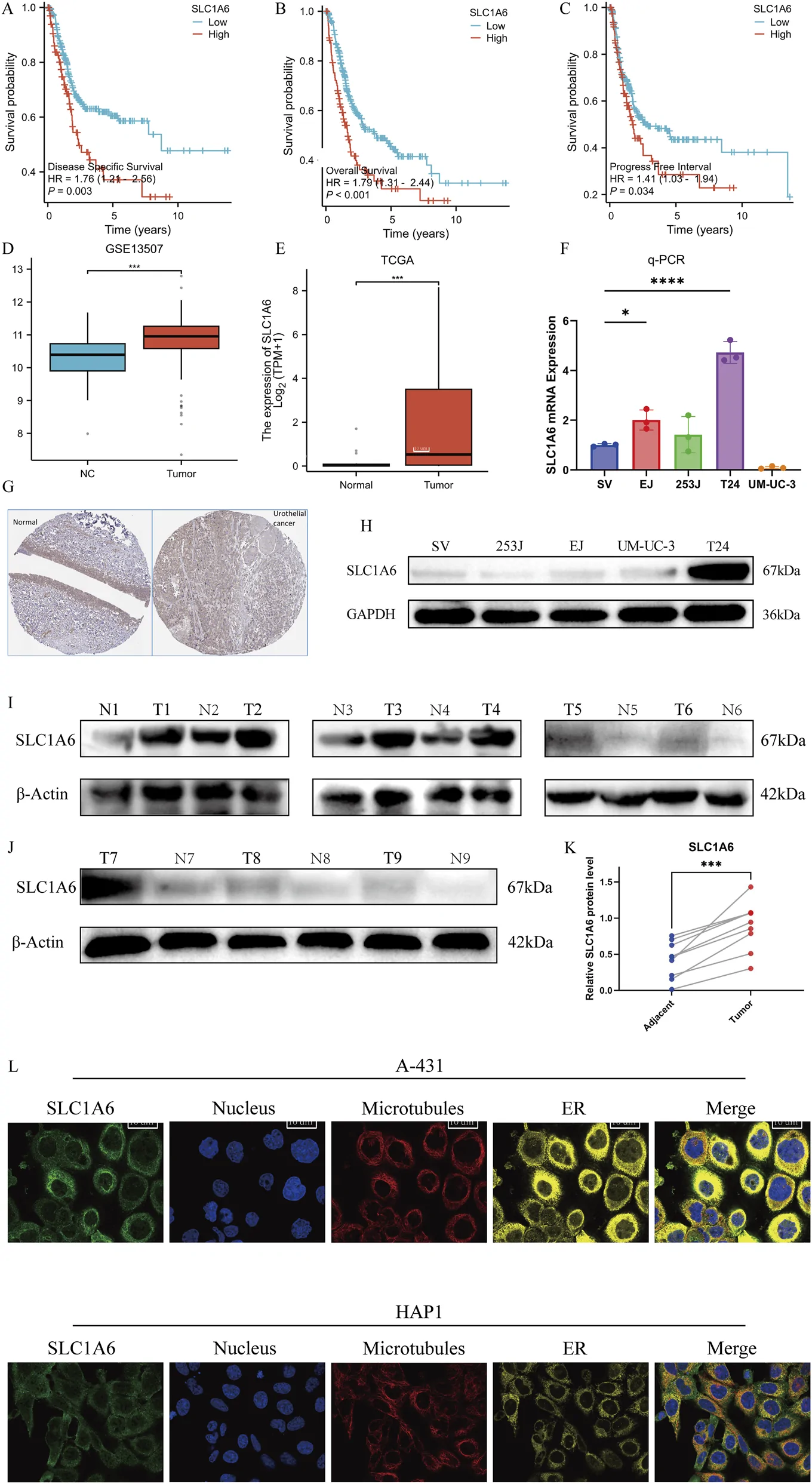
SLC1A6 is upregulated in bladder cancer and associated with poor prognosis. **(A–C)** Kaplan–Meier survival analyses of disease-specific survival (DSS), overall survival (OS), and progression-free interval (PFI) according to SLC1A6 expression in the TCGA-BLCA cohort. Hazard ratios (HRs), 95% confidence intervals (CIs), and P values are shown. **(D, E)** SLC1A6 expression in bladder cancer and normal bladder tissues in the GSE13507 and TCGA-BLCA cohorts. **(F)** qPCR analysis of SLC1A6 mRNA expression in SV normal urothelial cells and bladder cancer cell lines. **(G)** Representative Human Protein Atlas immunohistochemistry images showing SLC1A6 protein expression in normal bladder tissue and urothelial carcinoma. **(H)** Western blot analysis of SLC1A6 expression in SV cells and bladder cancer cell lines. **(I, J)** Western blot validation of SLC1A6 protein expression in paired bladder tumor tissues and adjacent non-tumor tissues. **(K)** Quantification of SLC1A6 protein expression in paired clinical samples. **(L)** Subcellular localization of SLC1A6 in A-431 and HAP1 cells based on Human Protein Atlas immunofluorescence images. Data are presented as mean ± SD where applicable. For panel F, each bladder cancer cell line was compared with SV cells using one-way ANOVA followed by Dunnett’s multiple comparisons test. *P < 0.05, ***P < 0.001, ****P < 0.0001.

### Elevated SLC1A6 expression is associated with unfavorable clinical outcomes in bladder cancer

To determine the clinical significance of SLC1A6, Kaplan–Meier survival analyses were performed. Patients with high SLC1A6 expression exhibited significantly worse disease-specific survival, overall survival, and progression-free interval than those with low SLC1A6 expression (Figures 3A–C), indicating that elevated SLC1A6 was consistently associated with poor clinical outcomes. Patients with high SLC1A6 expression exhibited significantly worse disease-specific survival, overall survival, and progression-free interval than those with low SLC1A6 expression (Figures 3A–C), indicating an association between elevated SLC1A6 expression and unfavorable clinical outcomes. In the univariate Cox analysis for overall survival, however, SLC1A6 expression did not reach statistical significance (Supplementary Figure S3C; HR = 1.02, P = 0.39). The multivariate model was therefore used to evaluate the adjusted association between SLC1A6 expression and survival rather than to indicate that SLC1A6 had been selected solely through univariate significance filtering.

### Pan-cancer analyses further supported the biological and clinical relevance of SLC1A6

To further characterize SLC1A6, immunofluorescence staining was examined in A-431 and HAP1 cells. SLC1A6 displayed a predominant cytoplasmic and membrane-associated distribution pattern, with partial overlap with intracellular structural compartments, supporting its potential functional involvement in cellular transport-related processes (Figure 3L). The extended correlation heatmap in Supplementary Figure S3B summarized the associations between SLC1A6 and the molecular and clinical features shown in the heatmap. This analysis was used as supporting exploratory evidence for the broader clinical and molecular relevance of SLC1A6.

### Exploratory pan-cancer analyses provide contextual evidence for SLC1A6 dysregulation

To avoid distracting from the bladder cancer focus, the broad pan-cancer analyses were moved to the Supplementary Material and interpreted as contextual evidence. Briefly, SLC1A6 showed cancer-type-specific associations with copy number variation, somatic mutation status, histologic grade, metastatic status, MSI, TMB, stemness-related indices, immune checkpoint-related genes, and immune/stromal infiltration estimates across TCGA cancer types (Supplementary Figures S4–S6). These findings suggest that SLC1A6 may be involved in diverse tumor-related molecular contexts, but they were not used as the main mechanistic evidence in this study. Therefore, the revised manuscript focuses primarily on bladder cancer-specific clinical associations, immune microenvironment features, single-cell localization, spatial enrichment, metabolic programs, and functional validation.

### SLC1A6 is associated with an immunosuppressive microenvironment in bladder cancer

To further explore the immunological relevance of SLC1A6 in bladder cancer, we systematically evaluated its association with molecular subtype, survival outcomes in immunotherapy-treated cohorts, and immune microenvironment characteristics. As shown in Figure 4A, SLC1A6 expression differed significantly among molecular subtypes in the TCGA-BLCA cohort, with the high-expression group being preferentially enriched in specific subtype categories (P = 0.002), suggesting a potential link between SLC1A6 and subtype-associated immune heterogeneity. Consistently, heatmap analysis further demonstrated distinct expression patterns of immune-related signatures and tumor microenvironment components between the SLC1A6 high- and low-expression groups (Figure 4B). To explore the relationship between SLC1A6 expression and clinical outcomes in immunotherapy-treated patients, survival analyses were performed in two public immunotherapy-treated cohorts. In both the Lauss cohort 2017 and the IMvigor210 cohort, patients with high SLC1A6 expression exhibited significantly poorer overall survival than those with low SLC1A6 expression (Figures 4C,D). These unadjusted analyses showed an association between SLC1A6-high status and unfavorable overall survival in the two immunotherapy-treated cohorts. However, because these analyses were based on unadjusted survival comparisons and the available public cohorts did not provide complete and harmonized immunotherapy-related covariates, including PD-L1, TMB, and response variables, these findings were not interpreted as evidence that SLC1A6 independently predicts immunotherapy resistance. We next characterized the relationship between SLC1A6 and immune cell infiltration. Correlation analysis based on immune infiltration algorithms revealed that SLC1A6 expression was broadly negatively associated with multiple antitumor immune cell populations, including CD8 T cells, cytotoxic cells, T cells, Th1 cells, NK CD56dim cells, and dendritic cells (Figure 4E). In line with this, enrichment analyses showed that the high-SLC1A6 group had significantly lower infiltration scores for T cells, Th1 cells, Treg cells, T-cell memory activated, T-cell CD4 naive, and T-cell CD4 memory resting compared with the low-expression group (Figures 4F–H, N–P). In addition, expression heatmaps further showed that SLC1A6 was negatively correlated with multiple immune checkpoint molecules, chemokines, chemokine receptors, MHC-related genes, and co-stimulatory/co-inhibitory immune regulators (Figures 4I–L), supporting a broad association between SLC1A6 upregulation and an immunologically suppressed tumor microenvironment. Similar trends were also observed in the CIBERSORT-based analysis, in which SLC1A6 was inversely correlated with several immune effector cell subsets but positively associated with limited stromal or resting immune populations (Figure 4M). Collectively, these findings indicate that high SLC1A6 expression is associated with an immune-deserted or immunosuppressed phenotype in bladder cancer and poorer survival outcomes in immunotherapy-treated cohorts.

**FIGURE 4:**
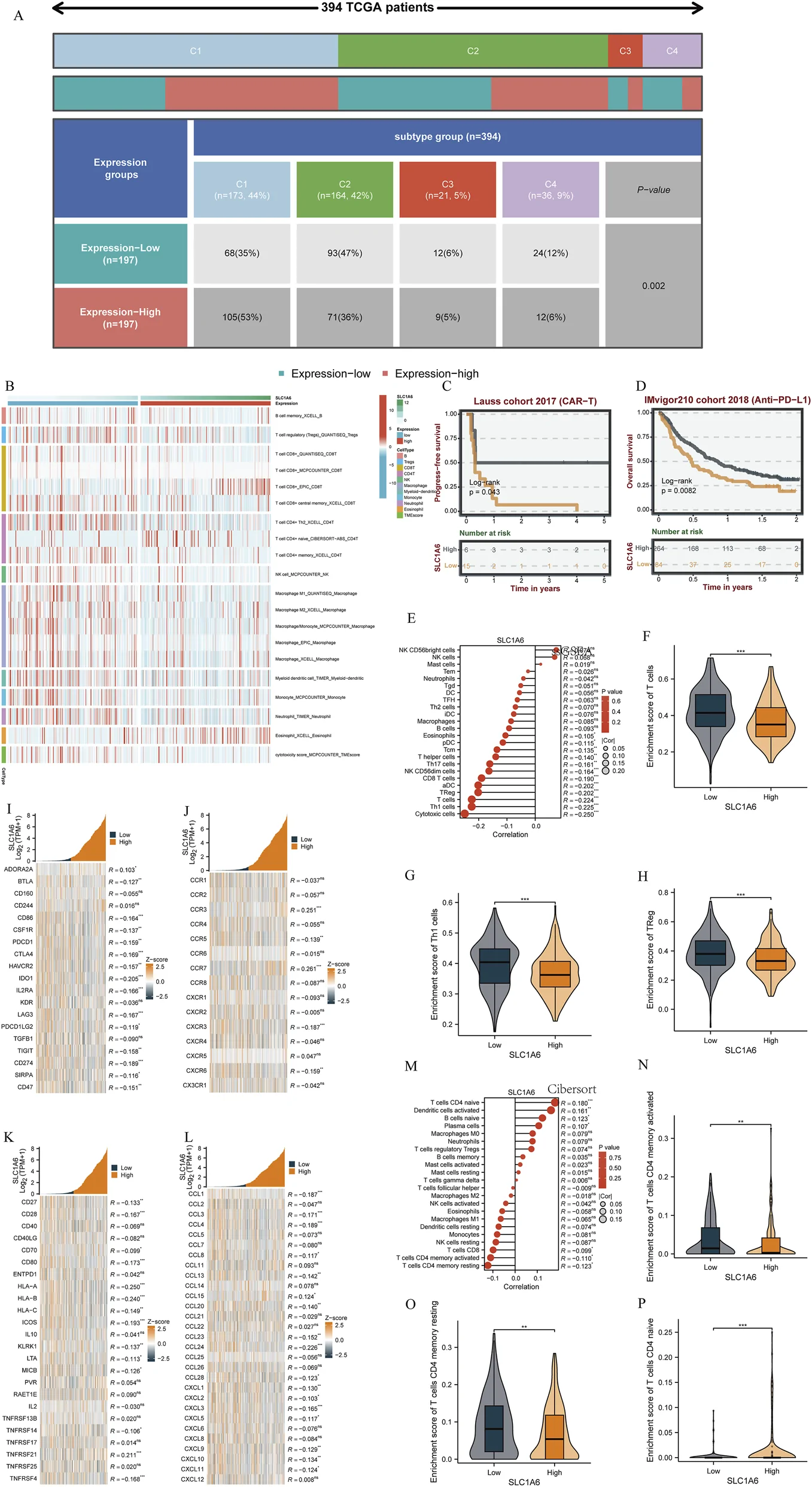
Association of SLC1A6 with the immune microenvironment and survival outcomes in immunotherapy-treated cohorts. **(A)** Distribution of SLC1A6 expression across molecular subtypes in the TCGA-BLCA cohort. **(B)** Heatmap showing differences in immune-related signatures and tumor microenvironment features between the SLC1A6 low- and high-expression groups. **(C, D)** Kaplan–Meier survival analyses of immunotherapy-treated patients in the Lauss cohort 2017 (CAR-T) and IMvigor210 cohort (anti-PD-L1) stratified by SLC1A6 expression. **(E, M)** Correlation analyses between SLC1A6 expression and immune cell infiltration estimated by different immune deconvolution algorithms **(F–H, N–P)** Comparison of enrichment scores for representative immune cell populations between the SLC1A6 low- and high-expression groups **(I–L)** Heatmaps showing the correlations of SLC1A6 with immune checkpoint genes, chemokines, chemokine receptors, MHC-related molecules, and other immune regulatory factors.

### SLC1A6 is associated with metabolic reprogramming in bladder cancer

To further characterize the biological relevance of SLC1A6 in bladder cancer, we first explored the metabolic differences associated with its expression. GSVA-based comparison between the SLC1A6 high- and low-expression groups in the TCGA-BLCA cohort revealed marked alterations in multiple metabolic pathways (Figure 5A). Among the positively enriched pathways in the high-expression group, alpha-linolenic acid metabolism, ether lipid metabolism, N-glycan biosynthesis, glycerophospholipid metabolism, and linoleic acid metabolism were prominently activated (Figures 5B–F), suggesting that elevated SLC1A6 was closely associated with lipid-related metabolic remodeling. In contrast, nucleotide metabolism, pyruvate metabolism, and one carbon pool by folate were enriched in the low-expression group (Figures 5G–I), indicating distinct metabolic states between the two groups. To further validate these observations, correlation analyses were performed between SLC1A6 expression and metabolism-related scores. SLC1A6 expression was negatively correlated with the Lasso score (Figure 5J) but positively correlated with the ferroptosis score, Bifurcation score, and Carnitine score (Figures 5K–M). These correlations were derived from transcriptomic signature-based scoring and were considered exploratory associations. In particular, the correlation with ferroptosis score was treated as hypothesis-generating and was not interpreted as evidence that SLC1A6 directly regulates ferroptosis. Supplementary Figure S1 showed that the pathways enriched in SLC1A6 were mainly involved in various KEGG metabolic pathways (including amino acid, glycolipid, xenobiotic and nucleotide metabolism, etc.) and several Hallmark gene sets related to metabolism, inflammatory response and interferon signaling.

**FIGURE 5:**
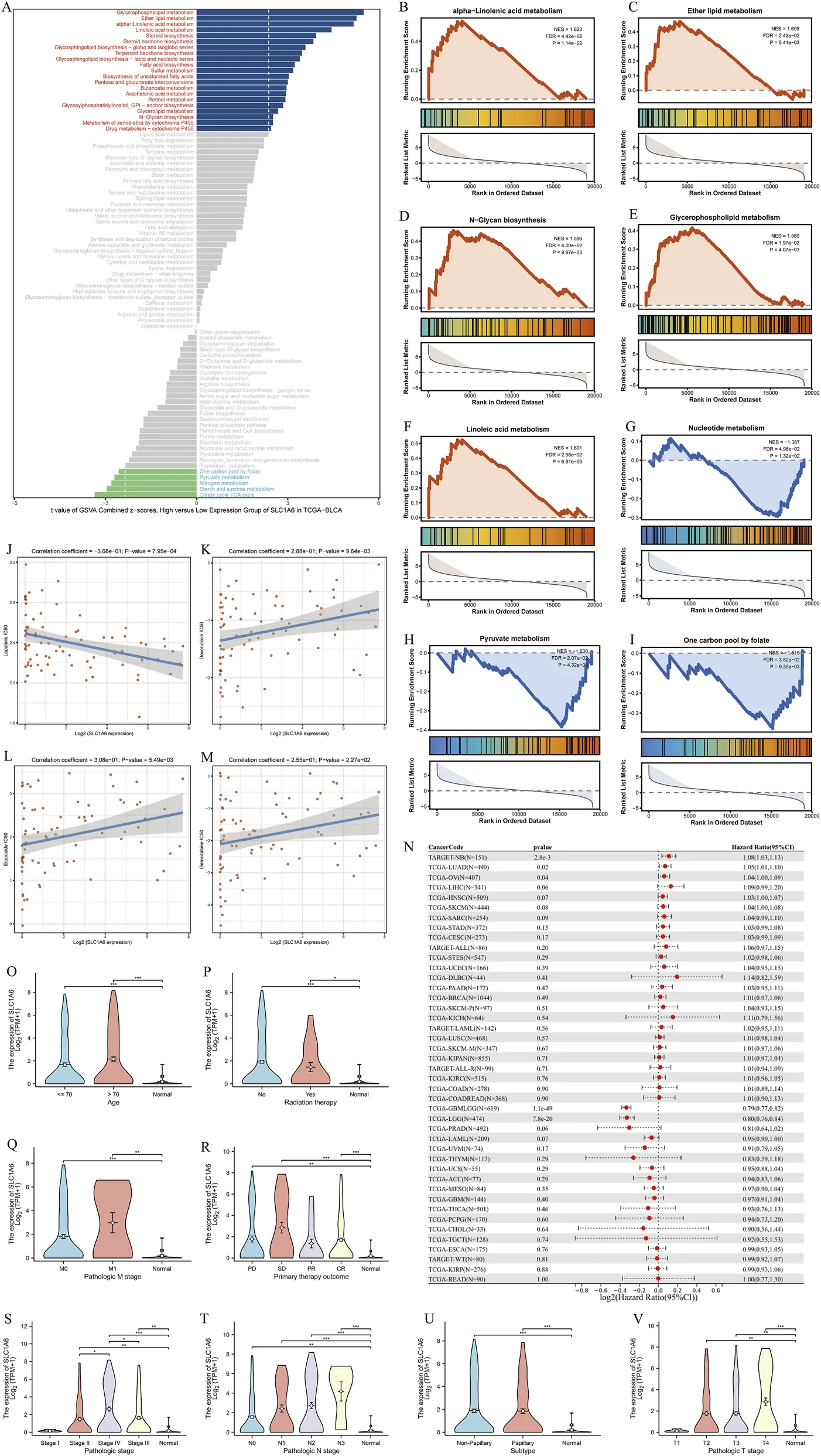
Metabolic characteristics, clinicopathological associations, and pan-cancer prognostic relevance of SLC1A6. **(A)** GSVA analysis showing differentially enriched metabolic pathways between the SLC1A6 high- and low-expression groups in the TCGA-BLCA cohort. **(B–F)** GSEA plots showing pathways positively enriched in the SLC1A6 high-expression group, including alpha-linolenic acid metabolism, ether lipid metabolism, N-glycan biosynthesis, glycerophospholipid metabolism, and linoleic acid metabolism. **(G–I)** GSEA plots showing pathways negatively associated with SLC1A6 expression, including nucleotide metabolism, pyruvate metabolism, and one carbon pool by folate. **(J–M)** Correlation analyses between SLC1A6 expression and metabolism-related scores, including Lasso score, ferroptosis score, Bifurcation score, and Carnitine score. **(N)** Pan-cancer Cox regression analysis of the prognostic value of SLC1A6 across multiple tumor types **(O–V)** Association of SLC1A6 expression with age, radiation therapy, pathologic M stage, primary therapy outcome, pathologic stage, pathologic N stage, subtype, and pathologic T stage in bladder cancer.

### Elevated SLC1A6 is associated with clinicopathological progression and therapeutic response

We next investigated the clinical relevance of SLC1A6 expression in bladder cancer. Stratified analyses showed that SLC1A6 expression differed significantly across multiple clinicopathological subgroups. Higher SLC1A6 expression was observed in patients aged >70 years compared with those aged ≤70 years (Figure 5O), and in patients receiving radiation therapy compared with those without radiation therapy (Figure 5P). In addition, SLC1A6 expression was significantly increased in patients with M1 stage relative to M0 stage (Figure 5Q), suggesting an association with distant metastasis. Consistent with this, SLC1A6 expression also varied significantly according to primary therapy outcome, with higher expression observed in patients with less favorable treatment responses (Figure 5R). Moreover, SLC1A6 expression increased progressively with advancing pathologic stage, pathologic N stage, and pathologic T stage (Figures 5S,T,V), supporting its close relationship with tumor progression and nodal involvement. Consistent with this stage-associated pattern, an additional stratified analysis showed that SLC1A6 expression was significantly higher in the MIBC group (T2–T4) than in the available NMIBC subgroup (T1) in the TCGA-BLCA cohort (Supplementary Figure S7A, P < 0.05). This finding further supports an association between elevated SLC1A6 expression and muscle-invasive disease. Differences were also observed between molecular or histological subtypes, with significantly altered expression between the papillary and non-papillary groups (Figure 5U). Taken together, these results suggest that elevated SLC1A6 expression is associated with more aggressive clinicopathological features and unfavorable therapeutic response in bladder cancer.

### Pan-cancer analysis supports the broader prognostic relevance of SLC1A6

To determine whether the clinical significance of SLC1A6 extended beyond bladder cancer, we further performed a pan-cancer Cox regression analysis across multiple tumor types. As shown in Figure 5N, SLC1A6 exhibited heterogeneous prognostic effects across cancers, acting as a risk factor in several malignancies while showing neutral or context-dependent effects in others. Although the magnitude and direction of association varied among tumor types, this analysis supports the notion that SLC1A6 may have broader prognostic relevance across human cancers. Similarly, PFI-based evaluation identified additional cancer types in which SLC1A6 expression exhibited prognostic relevance. The DFI analysis revealed distinct risk patterns in tumors for which disease-recurrence information was available. Assessment of DSS further confirmed that SLC1A6 expression contributed to survival differences in selected cancers, demonstrating cancer-type–specific prognostic relevance (Supplementary Figures S2A–C). Overall, these results indicate that SLC1A6 is not only associated with poor prognosis in bladder cancer but also exhibits pan-cancer prognostic significance, with differential risk patterns across human malignancies.

### Integrated single-cell transcriptomic analysis delineated the cellular landscape of bladder cancer

To analyze the cell-type-specific expression of SLC1A6 in bladder cancer, three independent single-cell RNA-sequencing datasets (GSE222315, GSE135337, GSE130001) were integrated for further analysis. Before batch correction, cells from different samples exhibited obvious dataset-specific separation in principal component space (Figure 6A). After Harmony-based integration, the batch effect was substantially reduced, and cells from different datasets showed improved overlap, indicating successful cross-cohort integration (Figure 6B). Unsupervised clustering identified 16 distinct cell clusters in the integrated bladder cancer single-cell atlas (Figure 6C). Cluster annotation was further performed according to canonical marker genes, as shown in the dot plot (Figure 6D), and the major cell populations were subsequently assigned as epithelial cells, fibroblasts, endothelial cells, macrophages, T cells, B cells, and plasma cells (Figures 6E,F). These results established a robust single-cell transcriptional framework for subsequent analyses of SLC1A6 expression and function in bladder cancer.

**FIGURE 6:**
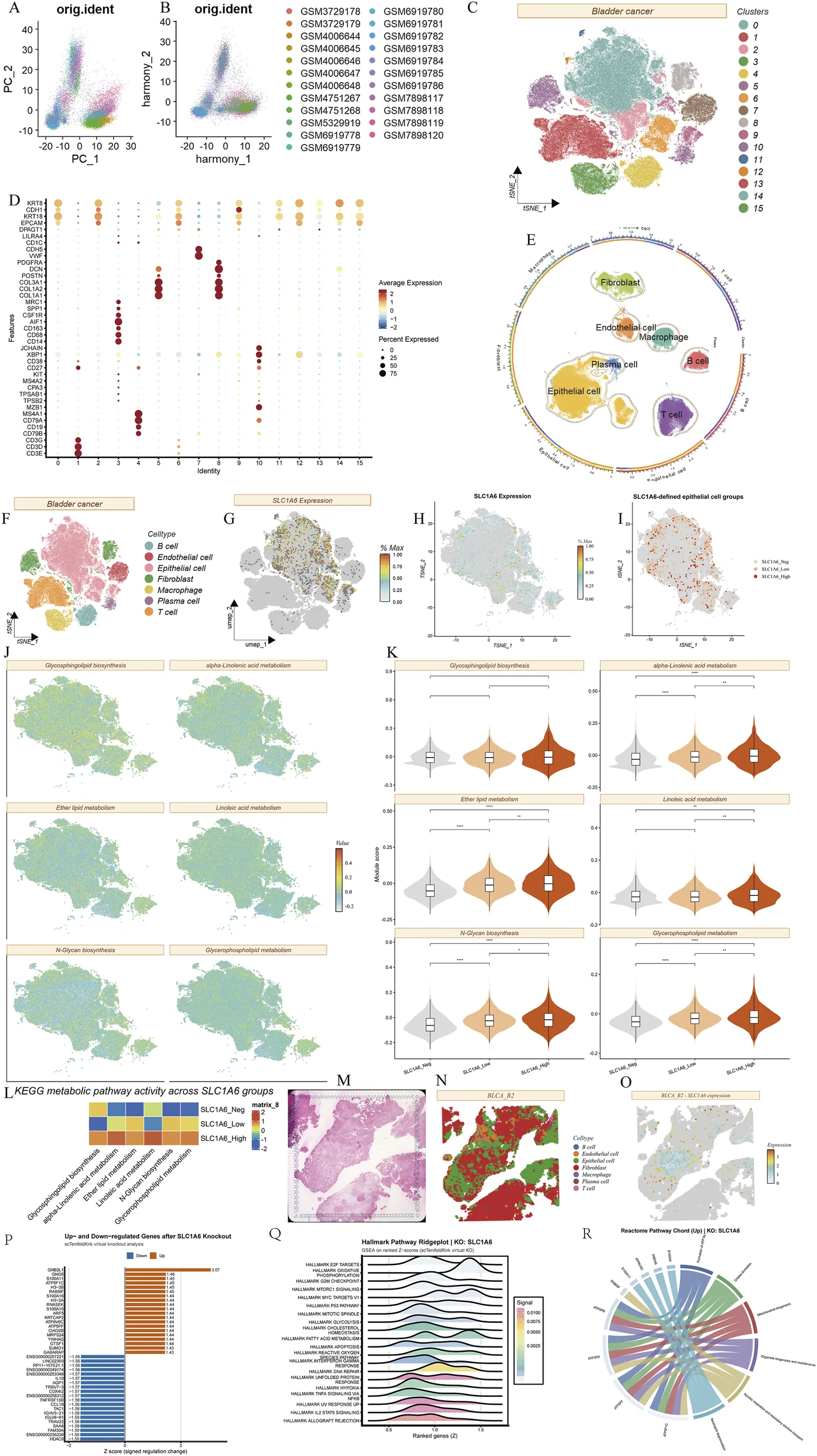
Single-cell, spatial, and perturbation analyses reveal the epithelial-specific expression and functional relevance of SLC1A6 in bladder cancer. **(A)** Distribution of cells from different samples before integration. **(B)** Distribution of cells after Harmony-based integration. **(C)** UMAP visualization of clustered cells in the integrated bladder cancer single-cell atlas. **(D)** Dot plot showing the expression of canonical marker genes across clusters. **(E, F)** Cell-type annotation and visualization of major cell populations in the integrated dataset. **(G, H)** Feature plots showing the single-cell expression pattern of SLC1A6 in bladder cancer. **(I)** UMAP visualization of SLC1A6-defined epithelial cell groups. **(J)** Single-cell projection of metabolic pathway activity associated with SLC1A6, including glycerophospholipid biosynthesis, alpha-linolenic acid metabolism, ether lipid metabolism, linoleic acid metabolism, N-glycan biosynthesis, and glycerophospholipid metabolism. **(K)** Violin plots comparing pathway activity among SLC1A6-negative, SLC1A6-low, and SLC1A6-high epithelial cell groups. **(L)** Heatmap summarizing KEGG metabolic pathway activity across SLC1A6-defined groups. **(M)** Representative histological image of the spatial transcriptomic section. **(N)** RCTD-based spatial deconvolution showing the distribution of major cell types in bladder cancer tissue. **(O)** Spatial expression map of SLC1A6 in the bladder cancer section. **(P)** Upregulated and downregulated genes identified after virtual knockout of SLC1A6. **(Q)** Ridgeline plot showing altered Hallmark pathway activities after virtual knockout of SLC1A6. **(R)** Reactome chord diagram summarizing pathway interactions associated with SLC1A6 perturbation.

### SLC1A6 was preferentially expressed in epithelial cells and defined a distinct epithelial subpopulation

We next investigated the cellular distribution of SLC1A6 in the integrated single-cell atlas. Feature visualization indicated that SLC1A6 expression was primarily concentrated in epithelial cells, with minimal expression in stromal or immune compartments (Figures 6G,H). To further assess epithelial heterogeneity associated with SLC1A6, epithelial cells were stratified according to SLC1A6 expression status, which identified SLC1A6-high, SLC1A6-low, and SLC1A6-negative epithelial cell groups (Figure 6I). These findings indicate that SLC1A6 is mainly expressed in the epithelial compartment of bladder cancer and may define a transcriptionally distinct epithelial subpopulation.

### Single-cell analyses validated the metabolic pathways associated with SLC1A6

Given the metabolic enrichment patterns observed in bulk transcriptomic analyses, we next examined whether these pathway alterations could also be recapitulated at single-cell resolution. Projection of pathway activity onto the single-cell map showed that several SLC1A6-associated metabolic programs, including glycerophospholipid biosynthesis, alpha-linolenic acid metabolism, ether lipid metabolism, linoleic acid metabolism, N-glycan biosynthesis, and glycerophospholipid metabolism, displayed distinct distribution patterns across the epithelial compartment (Figure 6J). Comparative analysis among SLC1A6-defined epithelial subgroups further demonstrated that these metabolic signatures were significantly elevated in SLC1A6-high epithelial cells relative to SLC1A6-low or SLC1A6-negative cells (Figure 6K). Consistently, the heatmap summarizing KEGG metabolic pathway activity across SLC1A6 groups also supported enhanced lipid- and glycan-related metabolic activity in the SLC1A6-high subgroup (Figure 6L). Together, these single-cell results further validated that SLC1A6 is closely associated with metabolic reprogramming, particularly in pathways related to lipid metabolism and glycosylation.

### Spatial transcriptomics and virtual knockout analyses further supported the spatial distribution and functional relevance of SLC1A6

To further validate the spatial localization of SLC1A6 in bladder cancer tissues, we analyzed the 10× Genomics Visium dataset GSE246011. Histological examination and spatial mapping demonstrated the overall tissue architecture (Figure 6M), while RCTD-based deconvolution using the integrated bladder cancer single-cell atlas as the reference revealed the spatial distribution of major cell types across the tumor section (Figure 6N). Spatial expression mapping showed that SLC1A6 signal was predominantly observed in epithelial-enriched regions identified by RCTD (Figure 6O), consistent with the single-cell expression pattern. Because this spatial localization was inferred computationally and was not independently validated by multiplex immunofluorescence or another orthogonal co-localization assay, it should be interpreted as supportive rather than definitive evidence of epithelial localization. To explore the potential functional impact of SLC1A6 perturbation, virtual knockout analysis was subsequently performed. The simulated loss of SLC1A6 resulted in widespread transcriptional alterations, including multiple upregulated and downregulated genes (Figure 6P). Hallmark pathway ridgeline analysis further indicated substantial pathway changes following SLC1A6 knockout (Figure 6Q), and Reactome chord analysis highlighted the signaling networks potentially influenced by SLC1A6 depletion (Figure 6R). Collectively, these findings support that SLC1A6 is spatially enriched in the epithelial compartment and may contribute to transcriptional and pathway-level programs relevant to bladder cancer progression.

### Virtual screening and molecular docking focused on SLC1A6 using structure-based methods

As summarized in Table 2, the virtual screening workflow started from 4,566 natural compounds in the L6000 Natural Product Library. LigPrep generated 14,194 ligand structures, of which 5,682 were retained after QikProp-based filtering. Hierarchical Glide docking further narrowed the candidates to 852 hits in the HTVS stage, 170 hits in the SP stage, and 132 top-ranked candidates in the XP stage for subsequent MM/GBSA-based prioritization. Among the screened compounds, ethyl rosmarinate (ER) showed the most favorable docking score, with a predicted binding energy of −10.56 kcal/mol, ranking first among the candidates (Figure 7A). Other top-ranked compounds included myricetin, orcinol glucoside, 4-O-p-coumaroylquinic acid, piceatannol, dihydrocurcumin, and curcumin, but their predicted binding affinities were weaker than that of ER. To provide an overview of the virtual screening results, the top 30 candidate compounds were visualized according to docking score. ER was clearly separated from the remaining candidates and displayed the strongest predicted binding affinity toward SLC1A6 (Figure 7B). This suggested that ER may have a favorable interaction profile with the selected SLC1A6 binding pocket. To further evaluate the robustness of compound prioritization, a dual-metric ranking strategy was applied by integrating docking score and MM/GBSA binding free energy. ER retained the best docking score and showed a favorable MM/GBSA ΔG bind value, supporting its stable predicted interaction with SLC1A6 (Figure 7C). Although several compounds, including 8-prenylnaringenin, dihydrocurcumin, and piperlongumine, exhibited relatively favorable MM/GBSA values, their docking scores were less optimal than that of ER. These findings indicate that ER remained the leading candidate when both docking affinity and binding free-energy estimation were considered. The relationship between molecular weight and docking affinity was next examined among the screened compounds. The scatter plot showed a general trend in which compounds with higher molecular weight tended to exhibit stronger predicted binding affinity. ER was highlighted as the rank 1 compound and showed the lowest docking score among the candidate molecules (Figure 7D). This result further supported ER as the most prominent candidate identified by the virtual screening workflow. Molecular docking visualization was then used to characterize the predicted binding mode between ER and SLC1A6. The global docking model showed that ER was positioned within a defined binding pocket of SLC1A6 (Figure 7E). Three-dimensional interaction analysis demonstrated that ER was surrounded by multiple amino acid residues, including ARG98, PRO188, ASN189, GLU192, LYS196, PHE198, PRO260, and ASN267 (Figure 7F). Two-dimensional ligand–receptor interaction mapping further revealed that ER formed several non-covalent interactions within the binding pocket, including conventional hydrogen bonds, hydrophobic contacts, alkyl interactions, π-related interactions, and van der Waals forces (Figure 7G). These interactions may contribute to the favorable predicted binding affinity between ER and SLC1A6.

**TABLE 2:** Summary of the structure-based virtual screening workflow for SLC1A6.

Screening step	Number retained	Selection criterion
Initial compound library	4,566 compounds	L6000 natural product library for HTS
LigPrep-generated ligand structures	14,194 structures	Generation of ionization states, stereoisomers, tautomers, and low-energy conformations
QikProp-filtered ligand structures	5,682 structures	ADME/T-related properties, drug-likeness, and chemical-space filtering
HTVS docking hits	852 structures	Top 15% of candidates ranked by docking score
SP docking hits	170 structures	Top 20% of candidates ranked by docking score
XP docking candidates	132 candidates	Top-ranked candidates after XP docking
Lead candidate	Ethyl rosmarinate	Best integrated docking/MM/GBSA ranking

**FIGURE 7:**
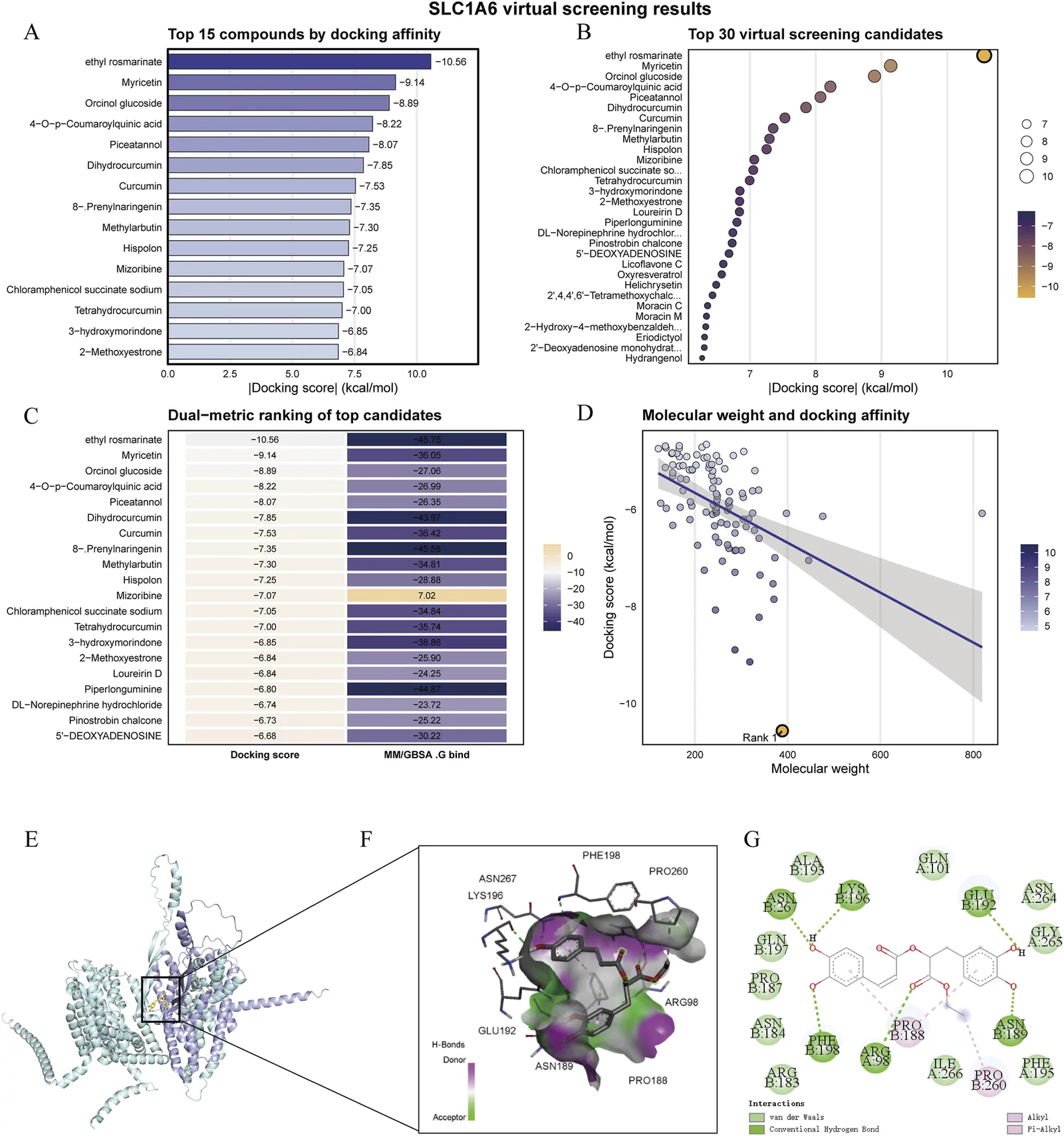
Structure-based virtual screening and molecular docking targeting SLC1A6. **(A)** Bar plot showing the top 15 candidate compounds ranked by docking affinity against SLC1A6. Ethyl rosmarinate (ER) showed the most favorable docking score among the screened compounds. **(B)** Bubble plot showing the top 30 virtual screening candidates ranked by docking score. Bubble size and color indicate the relative magnitude of predicted binding affinity. **(C)** Dual-metric heatmap of top candidate compounds integrating docking score and MM/GBSA ΔG bind values. Lower docking scores and more favorable binding free-energy values indicate stronger predicted ligand–receptor interactions. **(D)** Scatter plot showing the relationship between molecular weight and docking affinity among screened compounds. ER was highlighted as the rank 1 candidate with the strongest predicted binding affinity. **(E)** Global docking model showing the predicted binding location of ER within the SLC1A6 protein structure. The boxed region indicates the ligand-binding pocket selected for detailed visualization. **(F)** Three-dimensional interaction view of ER docked into the SLC1A6 binding pocket. Key surrounding residues are labeled, including ARG98, PRO188, ASN189, GLU192, LYS196, PHE198, PRO260, and ASN267. **(G)** Two-dimensional ligand–receptor interaction diagram showing the predicted non-covalent interactions between ER and SLC1A6, including conventional hydrogen bonds, hydrophobic contacts, alkyl interactions, π-related interactions, and van der Waals forces.

### ER suppresses malignant phenotypes across two bladder cancer cell lines

Functional validation was conducted in T24 bladder cancer cells to assess the biological impact of ER. First, we reassessed the cytotoxicity profile of ER using time-course CCK-8 assays in both T24 bladder cancer cells and SV-HUC-1 normal urothelial cells. Cells were treated with ER at 0, 0.625, 1.25, 2.5, 5, 10, 20, 40, or 80 μM for 24, 48, and 72 h. In T24 cells, ER reduced cell viability in a dose- and time-dependent manner, with higher concentrations and longer exposure times producing stronger inhibitory effects (Figure 8A). In SV-HUC-1 cells, ER also showed a dose-dependent inhibitory effect, but the calculated IC50 values were higher than those in T24 cells at all tested time points (Figure 8B; Table 3). Dose-response curve fitting showed that the IC50 values of ER in T24 cells were 27.05 μM at 24 h, 14.19 μM at 48 h, and 7.87 μM at 72 h. In SV-HUC-1 cells, the corresponding IC50 values were 32.35 μM, 21.63 μM, and 15.94 μM at 24, 48, and 72 h, respectively. The calculated selectivity indices were 1.20, 1.52, and 2.03 at 24, 48, and 72 h, respectively, suggesting that ER exerted relatively stronger growth-inhibitory effects in T24 cells than in normal urothelial cells. Importantly, 5 μM ER was below the calculated IC50 values in both cell lines at all tested time points and was therefore selected as the working concentration for subsequent phenotypic assays. Western blot analysis was then performed to determine whether ER affected SLC1A6 expression in T24 cells. Compared with the control group, ER treatment markedly reduced SLC1A6 protein expression, and densitometric quantification confirmed a significant decrease in relative SLC1A6 levels after ER exposure (Figures 8C,D). CETSA further showed that 5 μM ethyl rosmarinate altered the thermal stability profile of SLC1A6 compared with DMSO control, supporting cellular target engagement between ethyl rosmarinate and SLC1A6 in T24 cells (Figures 8E,F). These findings support cellular engagement between ER and SLC1A6, but do not establish that the subsequent phenotypic effects of ER are mediated specifically through SLC1A6. We next evaluated the phenotypic effects of ER under the selected working concentration. In the wound healing assay, comparable wound areas were observed between the control and ER-treated groups at 0 h. After 24 h, vehicle-treated cells showed more obvious wound closure, whereas ER-treated cells exhibited delayed wound closure. Quantitative analysis showed that ER treatment reduced the wound healing rate compared with the vehicle control group, suggesting decreased migratory capacity under the tested experimental conditions (Figures 8G,H). The effect of ER on clonogenic growth was further assessed using a colony formation assay. Compared with vehicle-treated cells, ER-treated cells formed fewer visible colonies, and quantitative analysis showed a lower colony number in the ER-treated group (Figures 8I,K). Transwell invasion assays also showed that ER-treated cells displayed fewer invaded cells crossing the membrane than vehicle-treated cells (Figures 8J,L). Together, these results suggest that ER treatment was associated with reduced migratory, clonogenic, and invasive phenotypes in T24 cells relative to vehicle control. Because these assays did not include established cytotoxic or anti-migratory reference inhibitors, the observed effects were interpreted as relative phenotypic changes under the selected experimental conditions rather than as direct pharmacological benchmarking of ER effect size. To determine whether these phenotypic effects were reproducible in an additional bladder cancer model, we further evaluated ER in UM-UC-3 cells. Dose-response viability analysis showed that 5 μM ER caused only a modest reduction in UM-UC-3 cell viability and was therefore retained as the working concentration for functional validation (Supplementary Figure S7B). In wound-healing assays, ER-treated UM-UC-3 cells exhibited significantly reduced wound closure compared with vehicle-treated cells at 24 h (Supplementary Figures S7C, F). ER treatment also significantly reduced colony formation (Supplementary Figures S7E, G) and the number of invaded cells in Transwell assays (Supplementary Figures S7D, H). These findings indicate that the inhibitory phenotypic effects of ER were reproducible in a second bladder cancer cell line and were therefore not restricted to T24 cells.

**FIGURE 8:**
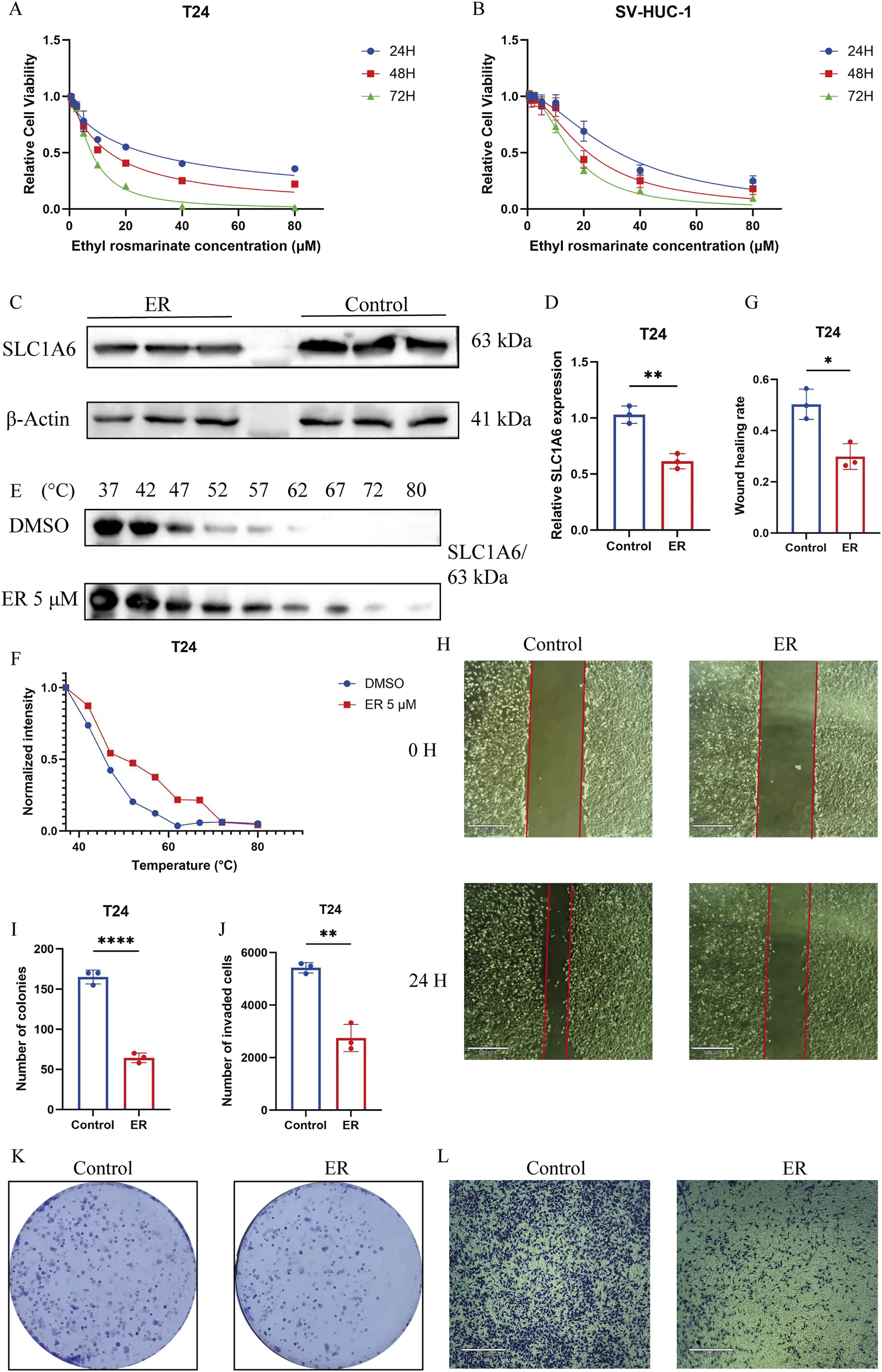
Experimental validation of ethyl rosmarinate in bladder cancer cells. **(A, B)** Time-course CCK-8 viability curves of T24 bladder cancer cells and SV-HUC-1 normal urothelial cells treated with different concentrations of ethyl rosmarinate (ER) for 24, 48, and 72 h. **(C, D)** Western blot analysis and quantification of SLC1A6 protein expression in T24 cells treated with ER or vehicle control. **(E, F)** Cellular thermal shift assay (CETSA) showing the thermal stability of SLC1A6 in T24 cells treated with DMSO or 5 μM ER. **(G, H)** Quantification and representative images of wound healing assays in control and ER-treated T24 cells. **(I, K)** Quantification and representative images of colony formation assays. **(J, L)** Quantification and representative images of Transwell invasion assays. Data are presented as mean ± SD from three independent biological replicates. ER, ethyl rosmarinate; DMSO, dimethyl sulfoxide. *P < 0.05, **P < 0.01, ****P < 0.0001.

**TABLE 3:** Time-dependent IC50 values of ethyl rosmarinate in T24 and SV-HUC-1 cells.

Time point	T24 IC50 (μM)	SV-HUC-1 IC50 (μM)	Selectivity index
24 h	27.05	32.35	1.20
48 h	14.19	21.63	1.52
72 h	7.87	15.94	2.03

## Discussion

This study presents a multi-omics analysis that identifies SLC1A6 as a novel tumor-enriched gene with significant clinical and biological relevance in bladder cancer progression. Through integrative analyses combining differential expression, WGCNA module–trait relationships, and cross-cohort validation in GSE13507 and TCGA-BLCA, SLC1A6 emerged as one of the most robust genes distinguishing tumor from normal tissue. The prognostic significance of SLC1A6 is reinforced by its consistent link to poorer overall, progression-free, and disease-specific survival outcomes. This observation supports existing evidence that solute carrier (SLC) family members are crucial in tumor growth and metabolic reprogramming, as previous studies have shown SLC transporters to be key regulators of nutrient acquisition and metabolic homeostasis in cancer (Fairman et al., 1995).

Although SLC1A6 is traditionally characterized as a high-affinity glutamate transporter expressed in cerebellar Purkinje neurons (Zhu et al., 2025), its involvement in cancer has remained largely unexplored. By leveraging pan-cancer datasets, our study revealed that SLC1A6 expression is dysregulated not only in bladder cancer but also across multiple malignancies, suggesting a broader biological spectrum beyond its established neuronal role. This extends the understanding of SLC transporters, particularly given prior reports that other glutamate transporters, such as SLC1A5 and SLC1A3, can drive metabolic flexibility, redox balance, and tumor proliferation (Shah et al., 2021; Shiau et al., 2024). The present findings thus suggest that SLC1A6 may function analogously to these transporters, contributing to the metabolic adaptations characteristic of aggressive urothelial carcinoma. The positive correlation between SLC1A6 expression and ferroptosis score was derived from transcriptomic signature-based scoring and should be regarded as a hypothesis-generating observation rather than mechanistic evidence. Although ethyl rosmarinate has antioxidant properties that could theoretically intersect with ferroptosis-related processes, the present study did not evaluate lipid peroxidation, intracellular iron accumulation, ferroptosis-related protein markers, or rescue by ferroptosis inhibitors. Therefore, no mechanistic link among SLC1A6, ethyl rosmarinate, and ferroptosis can be established from the current data. Future studies will be required to determine whether such a relationship exists.

The initial KEGG enrichment analysis identified several distinct biological themes among the intersecting bladder cancer candidate genes, including mismatch repair, lysine degradation, inositol phosphate metabolism, and multiple cancer- and signaling-related pathways. Mismatch repair was among the most prominent findings and may reflect altered DNA repair and genomic maintenance in the broader bladder cancer-associated gene set. Lysine degradation may indicate changes in amino acid metabolism and epigenetic regulation, while inositol phosphate metabolism and signaling-related pathways suggest additional metabolic and signaling complexity. Importantly, because these enrichment results were derived from the intersecting candidate gene set before SLC1A6-specific prioritization, they should not be interpreted as direct evidence that SLC1A6 regulates these pathways. Instead, they provide biological context for the candidate-gene landscape. Subsequent SLC1A6-centered analyses independently highlighted epithelial localization and lipid- and glycan-related metabolic programs. Thus, the different enrichment results likely represent complementary levels of analysis rather than mutually exclusive biological mechanisms.

Spatial transcriptomics provided contextual insight into the microanatomical distribution of SLC1A6. Using the GSE246011 Visium dataset and RCTD-based deconvolution with the integrated bladder cancer single-cell atlas as the reference, we observed that SLC1A6 expression was mainly localized to epithelial-enriched tumor regions rather than being diffusely distributed across the tumor microenvironment. These regions showed limited overlap with immune-rich or fibroblast-rich compartments, supporting the interpretation that SLC1A6 is predominantly associated with the tumor epithelial compartment. Spatially constrained metabolic programs have been increasingly recognized in solid tumors, with nutrient transporters serving as important mediators of local metabolic ecosystems. Our spatial findings therefore suggest that SLC1A6 may contribute to an epithelial niche-associated metabolic phenotype in bladder cancer (Zuschlag et al., 2025).

The clinical relevance of SLC1A6 was further underscored by its strong association with tumor burden, nodal status, metastatic spread, treatment outcomes, and multiple tumor behaviour indicators such as TMB, MSI, stemness scores, and immune infiltration patterns. Patients with higher SLC1A6 expression tended to exhibit more advanced disease, suggesting that SLC1A6 may be activated during tumor progression or selected for under microenvironmental pressure. Furthermore, pharmacogenomic correlations revealed that SLC1A6 expression was linked to differential sensitivity to several anticancer agents, implying that metabolic transporter expression may influence therapeutic response. Recent assay-development work has established methods for evaluating pharmacological inhibition of SLC1A3, SLC1A2, and SLC1A1 (Zuschlag et al., 2025). Previous work has also used single-cell-informed deconvolution to investigate resistance to immune checkpoint blockade in urothelial cancer (Wang et al., 2024).

Importantly, our findings were validated experimentally at the protein level. Western blot analysis of nine paired clinical samples demonstrated a significant upregulation of SLC1A6 in tumor tissues relative to adjacent normal tissues, aligning with transcriptomic and spatial data. Immunohistochemical staining from the HPA database also demonstrated epithelial localization of SLC1A6, supporting the conclusion that SLC1A6 is enriched specifically in the tumor epithelium. These findings, derived from diverse technologies such as bulk RNA-seq, single-cell reference modeling, spatial transcriptomics, pan-cancer profiling, and protein-level experiments, collectively highlight the robustness and biological plausibility of SLC1A6 as a tumor-intrinsic molecule in bladder cancer.

Despite the strengths of multi-layered validation and integrative analysis, several limitations warrant consideration. First, the present study is primarily observational; although the association between SLC1A6 and adverse prognosis is compelling, functional experiments are needed to determine whether SLC1A6 directly promotes tumorigenesis or reflects a broader metabolic adaptation. Second, while spatial transcriptomics provided high-resolution spatial localization, larger datasets are required to validate the consistency of SLC1A6 spatial patterns across different molecular subtypes of bladder cancer. In addition, although the concordance between single-cell and spatial transcriptomic analyses supports epithelial enrichment of SLC1A6, this localization was not independently confirmed by multiplex immunofluorescence or another cell-type-resolved protein-level co-localization assay. Therefore, the current spatial findings should be regarded as supportive rather than definitive evidence of epithelial localization. Although docking, CETSA, and ER-induced reduction of SLC1A6 protein expression support an interaction between ER and SLC1A6 at the computational and cellular levels, these data do not establish that SLC1A6 is the causal mediator of the anti-malignant effects of ER. Genetic loss-of-function or rescue experiments would be required to determine whether suppression of migration, clonogenic growth, and invasion depends on SLC1A6. Therefore, off-target or parallel effects of ER cannot be excluded in the present study. Although vehicle-controlled comparisons showed that ER treatment reduced migration, clonogenic growth, and invasion in both T24 and UM-UC-3 cells, the present validation remains limited to established bladder cancer cell lines. Further studies in additional molecular subtypes, organoids, and patient-derived models will be required to determine the broader generalizability of these effects. Future pharmacological studies should include appropriate reference agents, such as standard cytotoxic drugs for clonogenic assays and validated motility or invasion inhibitors for wound healing and Transwell assays, to better define the comparative effect size of ER. Although high SLC1A6 expression was associated with poorer overall survival in two immunotherapy-treated cohorts, these findings were derived from unadjusted Kaplan–Meier analyses and should be interpreted as exploratory survival associations rather than evidence of an independent predictive biomarker. Complete and harmonized covariates, including PD-L1 status, TMB, tumor mutational signatures, treatment response, and other relevant clinical predictors, were not uniformly available across the public cohorts; therefore, a reliable multivariable adjustment could not be performed. Future studies in larger immunotherapy-treated cohorts with comprehensive biomarker annotation will be required to determine whether SLC1A6 has independent predictive value. Although TCGA and GTEx samples were obtained from the uniformly reprocessed Toil dataset to reduce computational batch effects, residual differences related to tissue acquisition, library preparation, cohort composition, and other pre-analytical factors cannot be completely excluded. Therefore, the TCGA–GTEx comparisons should be interpreted as supportive pan-cancer evidence rather than definitive cross-cohort validation.

This study highlights SLC1A6 as a novel and clinically significant tumor-intrinsic molecule in bladder cancer, enhancing the understanding of solute carrier transporters in oncology. The consistent overexpression of SLC1A6 across transcriptomic, spatial, and protein-level datasets suggests that it may contribute to tumor metabolic reprogramming, disease progression, and therapeutic responsiveness. These findings lay the groundwork for future studies to clarify the biological role of SLC1A6 and assess its potential as a prognostic biomarker or therapeutic target in urothelial carcinoma.

## Data Availability

The original contributions presented in the study are included in the article/Supplementary Material, further inquiries can be directed to the corresponding authors.

## Supplementary material

SUPPLEMENTARY FIGURE S1 Pathway-level associations of SLC1A6 across bladder cancer-related cohorts.Bubble plot showing the association between SLC1A6 expression and pathway activity scores across multiple bladder cancer cohorts. Dot color represents the normalized enrichment score (NES), and dot size represents −log10(adjusted P value).

SUPPLEMENTARY FIGURE S2 Pan-cancer prognostic analysis of SLC1A6.Forest plots showing the association between SLC1A6 expression and survival outcomes across TCGA cancer types. Hazard ratios (HRs), 95% confidence intervals (CIs), and P values are shown for each cancer type.

SUPPLEMENTARY FIGURE S3 Extended validation, prognostic modeling, and pan-cancer expression analyses of SLC1A6. **(A)** Primer amplification efficiency validation for SLC1A6 and GAPDH using five-point serially diluted cDNA standard curves. Ct values were plotted against log_10_(relative cDNA input), and amplification efficiency was calculated from the slope of the linear regression curve. **(B)** Correlation analysis of SLC1A6 with the molecular and clinical features shown in the heatmap. Color indicates the direction of correlation, and bubble size indicates the magnitude or statistical significance of the association. **(C, D)** Univariate and multivariate Cox regression analyses evaluating the association of SLC1A6 and clinicopathological variables with overall survival in the TCGA-BLCA cohort. Hazard ratios (HRs), 95% confidence intervals (CIs), and P values are shown. **(E)** Risk distribution and clinicopathological annotation of patients included in the prognostic model. **(F)** Nomogram constructed using SLC1A6 expression and clinicopathological variables to estimate 1-, 2-, and 3-year overall survival probability. **(G)** Calibration curves evaluating the agreement between nomogram-predicted and observed overall survival probabilities at 1, 2, and 3 years. **(H, I)** Pan-cancer expression analysis of SLC1A6 based on TCGA combined with GTEx normal tissues and TCGA tumor-normal comparisons. TCGA-BLCA, The Cancer Genome Atlas bladder cancer cohort; TCGA, The Cancer Genome Atlas; GTEx, Genotype-Tissue Expression; HR, hazard ratio; CI, confidence interval; Ct, cycle threshold. ns, not significant; *P < 0.05, **P < 0.01, ***P < 0.001, ****P < 0.0001.

SUPPLEMENTARY FIGURE S4 Association of SLC1A6 expression with genomic alterations and clinicopathologic features across cancers. **(A)** Violin plots showing SLC1A6 expression across copy number variation (CNV) categories (neutral, gain, loss) in TCGA pan-cancer cohorts. **(B)** Violin plots comparing SLC1A6 expression between wild-type (WT) and mutated (Mut) groups based on somatic single-nucleotide variation (SNV) status. **(C)** Violin plots of SLC1A6 expression across histologic grades (G1–G4) in cancers with available grade information. **(D)** Violin plots comparing SLC1A6 expression between non-metastatic (M0) and metastatic (M1) tumors. Asterisks indicate statistically significant differences between groups.

SUPPLEMENTARY FIGURE S5 Association of SLC1A6 expression with genomic instability, tumor stemness, immune regulation, and diagnostic performance. **(A)** Pearson correlation coefficients between SLC1A6 expression and microsatellite instability (MSI) across TCGA cancer types. **(B)** Correlation between SLC1A6 expression and tumor mutation burden (TMB). **(C)** Correlation between SLC1A6 and DNA methylation–based stemness index (DNAss). **(D)** Correlation between SLC1A6 and RNA expression–based stemness index (RNAss). **(E)** Correlation matrix showing relationships between SLC1A6 and immune checkpoint–related genes. **(F–K)** ROC curves illustrating the diagnostic performance of SLC1A6 in selected cancers (ESCA, GBM, GBMLGG, KICH, PRAD, BLCA), with corresponding AUC values and confidence intervals.

SUPPLEMENTARY FIGURE S6 Pan-cancer immune infiltration associations of SLC1A6 expression. **(A)** xCell-derived correlation heatmap showing associations between SLC1A6 expression and 64 immune/stromal cell infiltration scores across TCGA cancer types. **(B)** Correlation heatmap between SLC1A6 expression and four immunophenoscore (IPS) components, including effector cells (EC), suppressor cells (SC), antigen-presenting machinery (APM), and checkpoints (CP). **(C)** MCPcounter-based correlations between SLC1A6 and immune/stromal population abundance scores across multiple cancer types.Red/green colors represent positive or negative correlations, respectively, and triangle size indicates statistical significance.

SUPPLEMENTARY FIGURE S7 Additional clinicopathological analysis and functional validation of ethyl rosmarinate in UM-UC-3 bladder cancer cells. **(A)** Comparison of SLC1A6 expression between non-muscle-invasive bladder cancer (NMIBC; pathologic T1) and muscle-invasive bladder cancer (MIBC; pathologic T2–T4) in the TCGA-BLCA cohort. **(B)** Relative cell viability of UM-UC-3 cells treated with the indicated concentrations of ethyl rosmarinate (ER) for 24 h, supporting the selection of 5 μM ER for subsequent functional assays. **(C)** Representative wound-healing images of UM-UC-3 cells treated with vehicle control or 5 μM ER at 0 and 24 h. Red lines indicate the wound boundaries. **(D)** Representative images of Transwell invasion assays showing invaded UM-UC-3 cells following vehicle or 5 μM ER treatment. **(E)** Representative colony-formation images of UM-UC-3 cells treated with vehicle or 5 μM ER. **(F)** Quantification of the wound-healing rate at 24 h. **(G)** Quantification of colony numbers in the colony-formation assay. **(H)** Quantification of invaded cells in the Transwell invasion assay. Data are presented as mean ± SD where applicable. Statistical comparisons between two experimental groups were performed using Student’s t-test, whereas the comparison between NMIBC and MIBC in panel **(A)** was performed using the Wilcoxon rank-sum test. *P < 0.05, **P < 0.01, ****P < 0.0001. ER, ethyl rosmarinate; NMIBC, non-muscle-invasive bladder cancer; MIBC, muscle-invasive bladder cancer; TCGA-BLCA, The Cancer Genome Atlas bladder cancer cohort.
